# Clonal derivation of white and brown adipocyte progenitor cell lines from human pluripotent stem cells

**DOI:** 10.1186/s13287-018-1087-7

**Published:** 2019-01-08

**Authors:** Michael D. West, Ching-Fang Chang, Dana Larocca, Jie Li, Jianjie Jiang, Pamela Sim, Ivan Labat, Karen B. Chapman, Kari E. Wong, James Nicoll, Michael J. Van Kanegan, Aubrey D. N. J. de Grey, Igor O. Nasonkin, Andreas Stahl, Hal Sternberg

**Affiliations:** 1 AgeX Therapeutics, Inc., 1010 Atlantic Ave, Alameda, CA 94501 USA; 2 Johns Hopkins University, Baltimore, MD 21218 USA; 3 University of California, Berkeley, CA 94720 USA; 4 Metabolon Inc., Morrisville, NC 27560 USA; 5 Zen-Bio, Inc., Research Triangle Park, NC 27709 USA; 6 SENS Research Foundation, Mountain View, CA 94041 USA; 7 BioTime, Inc., Alameda, CA 94501 USA

## Abstract

**Background:**

The role of brown fat in non-shivering thermogenesis and the discovery of brown fat depots in adult humans has made it the subject of intense research interest. A renewable source of brown adipocyte (BA) progenitors would be highly valuable for research and therapy. Directed differentiation of human pluripotent stem (hPS) cells to white or brown adipocytes is limited by lack of cell purity and scalability. Here we describe an alternative approach involving the identification of clonal self-renewing human embryonic progenitor (hEP) cell lines following partial hPS cell differentiation and selection of scalable clones.

**Methods:**

We screened a diverse panel of hPS cell-derived clonal hEP cell lines for adipocyte markers following growth in adipocyte differentiation medium. The transcriptome of the human hES-derived clonal embryonic progenitor cell lines E3, C4ELS5.1, NP88, and NP110 representing three class of definitive adipocyte progenitors were compared to the relatively non-adipogenic line E85 and adult-derived BAT and SAT-derived cells using gene expression microarrays, RT-qPCR, metabolic analysis and immunocytochemistry. Differentiation conditions were optimized for maximal *UCP1* expression.

**Results:**

Many of the differentiated hEP cell lines expressed the adipocyte marker, *FAPB4*, but only a small subset expressed definitive adipocyte markers including brown adipocyte marker, *UCP1*. Class I cells (i.e., E3) expressed *CITED1*, *ADIPOQ*, and *C19orf80* but little to no *UCP1*. Class II (i.e., C4ELS5.1) expressed *CITED1* and *UCP1* but little *ADIPOQ* and *LIPASIN*. Class III (i.e., NP88, NP110) expressed *CITED1*, *ADIPOQ*, *C19orf80*, and *UCP1* in a similar manner as fetal BAT-derived (fBAT) cells. Differentiated NP88 and NP110 lines were closest to fBAT cells morphologically in adiponectin and uncoupling protein expression. But they were more metabolically active than fBAT cells, had higher levels of 3-hydroxybutyrate, and lacked expression of fetal/adult marker, *COX7A1*. The hEP BA progenitor lines were scalable to 17 passages without loss of differentiation capacity and could be readily rederived.

**Conclusions:**

Taken together, these data demonstrate that self-renewing adipocyte progenitor cells can be derived from hES cells and that they are functionally like BAT cells but with unique properties that might be advantageous for basic research and for development of cell-based treatments for metabolic diseases.

**Electronic supplementary material:**

The online version of this article (10.1186/s13287-018-1087-7) contains supplementary material, which is available to authorized users.

## Background

Obesity is the result of an undesirable imbalance of energy intake relative to expenditure. Cellular components regulating this balance include white adipose tissue (WAT), capable of storing energy primarily in the form of triglycerides, and brown adipose tissue (BAT) that differs from WAT in its potential to expend energy through uncoupled oxidative phosphorylation (OXPHOS) resulting in thermogenesis. In numerous mammalian species, brown adipocytes are believed to contribute to cold-adapted non-shivering thermogenesis through the expression of uncoupling protein-1 *(UCP1)*, a gene encoding an integral component of the mitochondrial inner membrane. Until recently, it was commonly believed that BAT was present primarily in hibernating animals and in newborn humans, but absent or without function in the adult human. The identification of BAT in adult humans [1] combined with the reported inverse correlation of BAT content with age [2], obesity [3], and type II diabetes [4] has stimulated research into possible modalities aimed at increasing BAT activity to restore energy balance and at potentially treating disorders associated with metabolic syndrome. Proposed therapeutic strategies include the stimulation of adrenergic pathways to activate existing tissue in vivo with PPAR-ɣ activators such as the thiazolidinediones [5], introduction of growth factors such as FGF21 [6], the use of adipokines released by BAT cells such as adiponectin [7] and lipasin (betatrophin), or alternatively the direct transplantation of BAT cells [8].

Human pluripotent stem (hPS) cells such as human embryonic stem (hES) or induced pluripotent stem (iPS) cells have the potential to provide a scalable source of all human somatic cell types [9] including brown adipose (BA) cells [10–13]. Early studies on hPS cell differentiation to BA cells required gene transfer [11] while more recent studies have shown differentiation to BA cells without gene transfer [10, 12, 13]. However, most reported protocols including those for hPS differentiation to BA progenitors cells lead to relatively impure populations of cells that lack the purity or site specificity required for the manufacture of human clinical-grade therapeutics. Moreover, scaling at the pluripotent cell stage is not as cost-effective as conventional cell scale-up. We have developed a method of generating purified and site-specific somatic cell types through the propagation of hPS cell-derived clonal human embryonic progenitor cell lines to overcome current issues of purity and scale [14]. We designated these cultures as human “embryonic progenitor” cells (hEP cells) due to their ability to self-renew under selected culture conditions, their persistent expression of embryonic developmental stage gene markers, and their lack of fetal/adult gene markers such as *COX7A1* that are preferentially expressed in cells that have traversed the embryonic-fetal transition [15]. The hEP cell lines also typically display limited lineage potential having lost pluripotency markers and pluripotent functionality.

In our initial characterization of approximately 200 hEP lines, we reported that they were often capable of robust expansion and displayed a diversity of > 140-fold distinct cell types [14]. Due to the clonal nature of these lines, the cells show site-specific markers such as homeobox genes that facilitate the identification of the lines as precursors to specific embryonic anlagen. For example, at least seven distinct osteochondral progenitor cell types could be expanded, as well as progenitors of cranial neural crest capable of differentiation into cellular components of the choroid plexus [16, 17]. Comparable fate space screening using HyStem-4D bead arrays routinely leads to highly reproducible results [18]. HyStem-C is currently being used in a clinical trial as an extracellular matrix for cell-assisted lipotransfer.

In an effort to identify white and brown adipocyte progenitors from our library of hEP cell lines that were capable of differentiation in HyStem-C, we screened a diverse panel hEP cell lines in HyStem-4D bead arrays under adipogenic differentiation conditions. We identified a subset of hEP cell lines that expressed definitive white and brown adipocyte gene markers, some of which were functionally similar to fBAT cells based on lipid accumulation, mitochondrial content, and metabolic and metabolomic characterization. However, embryonic BA differed from fBAT having higher metabolism, high β-hydroxybutyrate accumulation, and lacking *COX7A1* expression. We also identified optimal conditions for differentiation to BA in HyStem-C. The clonally pure adipocyte progenitor cells described here could facilitate in vitro models of human WAT vs. BAT cell differentiation not previously achievable with heterogeneous differentiation protocols and provide the basis for developing cell-based therapy for metabolic diseases.

## Results

### Selection of adipogenic lines from a panel of hES derived-progenitor cell lines

**Fig. 1:**
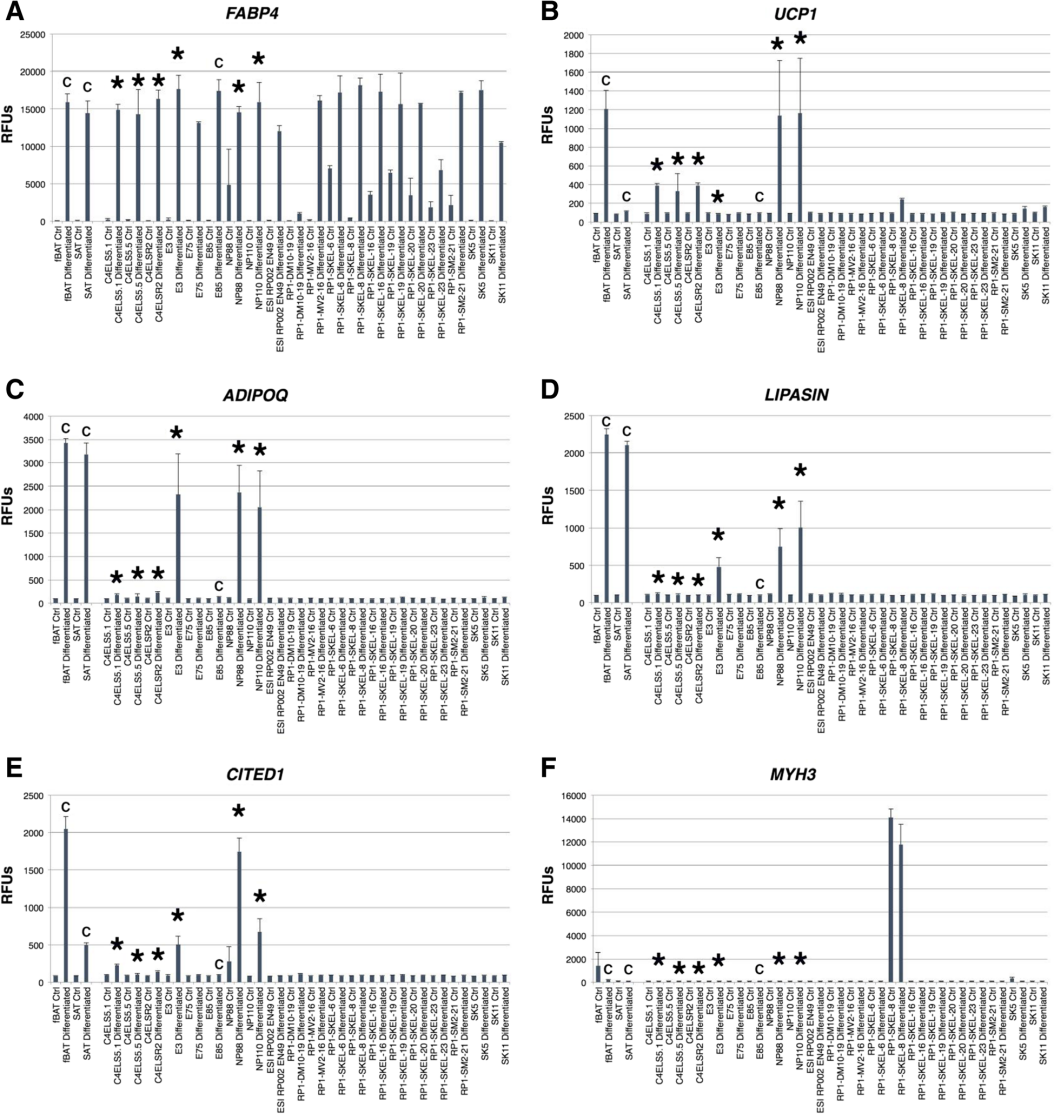
**a**–**f** Screening diverse cell types for BAT gene expression markers. **a** *FABP4*, **b** *UCP1*, **c** *ADIPOQ*, **d** *LIPASIN*, **e** *CITED1*, **f** *MYH3*, hES cell-derived clonal embryonic progenitor cell lines were analyzed by Illumina bead array-based gene expression for select adipocyte and BAT gene expression markers in the undifferentiated state and for 5 days in quiescence inducing conditions (Ctrl) or following adipogenic differentiation for 14 days in HyStem-C with BMP4, rosiglitazone, T3, and CL316243 (BMP4, Rosi, T3, CL) (differentiated). (C) Controls include adult BAT and SAT-derived preadipocytes and the clonal progenitor cell line E85 lacking the ability to differentiate into definitive adipocytes. Differentiated cells are identified as having definitive adipocyte markers. Values are shown as relative fluorescence units (RFUs) and represent mean values of two or more biological replicates. (RFU values < 130 considered background signal). (Error bars represent standard deviation)

In an effort to identify adipocyte progenitor cell lines from our library of hEP cell lines [14], we initially screened approximately 100 lines under control and adipogenic differentiation conditions (BMP4, Rosi, T3, CL; see the “Materials and methods” section). We encapsulated the cells in a collagen-hyaluronic acid matrix (HyStem-4D bead array) for the selection of lines that could differentiate in a biocompatible matrix that has been approved for use in human clinical studies [18]. Representative Illumina array transcriptomic data from 20 hEP lines, fBAT, and SAT (subcutaneous adult adipose tissue-derived cells) controls are shown in Fig. 1 and Additional file 1: Table S1. While most lines responded to the adipogenic differentiation conditions by markedly upregulating the expression of the commonly used adipocyte marker *FABP4*, a smaller subset responded with an up-regulation of a combination of the more definitive markers *UCP1*, *CITED1*, *ADIPOQ*, or *LIPASIN (BETATROPHIN*, *C19orf80).* The line E3 (class I) expressed *CITED1*, *ADIPOQ*, and *C19orf80* but low to undetectable levels of *UCP1.* The lines C4ELS5.1, C4ELS5.5, and C4ELSR2 (class II) expressed *CITED1* and *UCP1* but relatively low levels of *ADIPOQ* and *LIPASIN*. While the line RP1-SKEL-8 showed an induction of *UCP1*, it also strongly expressed skeletal myoblast markers such as the embryonic muscle marker *MYH3* and did not express the definitive adipocyte markers *CITED1*, *ADIPOQ*, or *LIPASIN*. However, the lines NP88 and NP110 (class III) expressed *CITED1*, *ADIPOQ*, *C19orf80*, and *UCP1* similar to cultured fetal BAT-derived cells*.* We therefore performed additional functional characterization of these two hEP cell lines.

### Comparison of selected hEP lines with fBAT and SAT cells by gene expression analysis

**Fig. 2:**
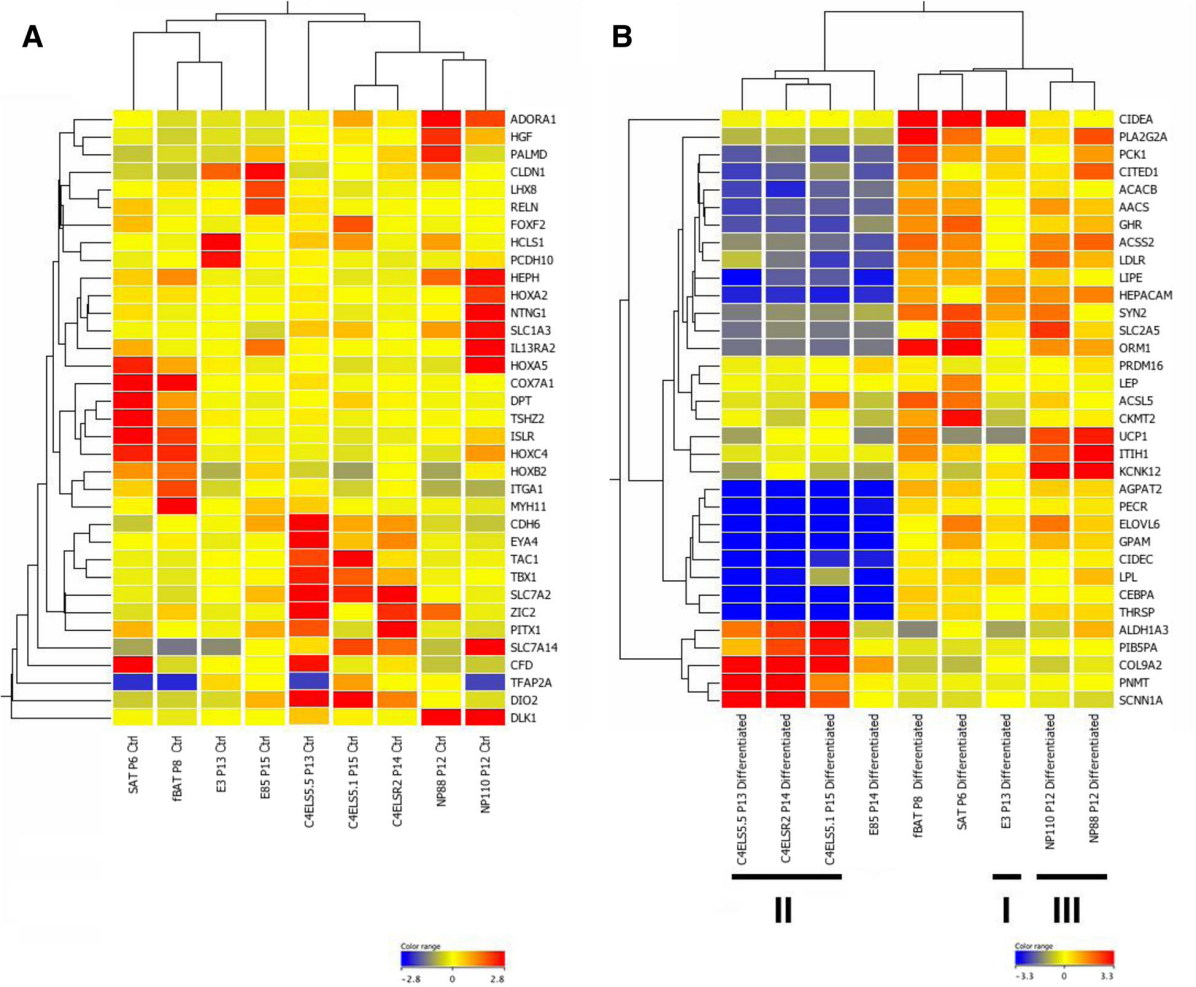
Heat maps of gene expression in select cell types in control and differentiated state. **a** Heat map of genes differentially expressed in the undifferentiated state. **b** Genes upregulated during differentiation and differentially expressed in the cell types. Clustering in **a** and **b** was performed on entire Illumina gene expression dataset. Color keys represent centered log_2_ values. Roman numerals designate class of clonal adipose progenitors

The Illumina bead array-generated transcriptomes of the lines E3, C4ELS5.1, C4ELS5.5, C4ELSR2, NP88, and NP110 showing induction of definitive adipocyte markers together were compared to adult fBAT and SAT controls in both the undifferentiated as well as the differentiated. The E85 line was used as a non-adipogenic hEP cell line control. In the undifferentiated state, clonal progenitor cell line E3 clustered most closely with fBAT cells (preadipocytes) as illustrated in a heat map of 34 genes of interest (Fig. 2a and Additional file 2: Table S2). Both E3 and fBAT expressed site-specific marker *HOXA5*, while E3 differed from fBAT and SAT preadipocytes in the expression of the cell adhesion gene *PCDH10* and the lack of expression of the fetal-adult marker *COX7A1* [19]. The cell lines C4ELS5.5, C4ELS5.1, and C4ELSR2 expressed relatively high levels of the transcriptional regulator *EYA4*, deiodinase-2 (DIO2), and the cationic amino acid transporter *SLC7A2*, but differed from each other in the varied expression of the site-specific markers *FOXF2* and *ZIC2*. The lines NP88 and NP110 clustered closely together sharing expression of *ADORA1*, *HEPH*, and *DLK1*, but differed from each other in that NP110 but not NP88 expressed *NTNG1, IL13RA2*, and the distal *HOX* gene markers *HOXA2* and *HOXA5*. Delta-like 1 homolog *(DLK1)* (also known as Preadipocyte factor 1 (*PREF1*)) is reported to be a major regulator of adipocyte replication and differentiation [20] as opposed to skeletal muscle differentiation. A polymorphism in the gene has been associated with the callipyge phenotype characterized by decreased adiposity and muscle hypertrophy [21].

Under adipogenic differentiation conditions, the fBAT, SAT, E3, NP88, and NP110 lines clustered together sharing the induction of adipocyte markers such as *PCK1*, *ACSL5*, *ACSS2*, *AGPAT2*, *CEBPA*, *HEPACAM*, *LDLR*, *LIPE*, *ORM1*, *PECR*, and *THRSP* as shown in the heatmap of a subset of markers in Fig. 2b and Additional file 2: Table S2. In addition, the phosphofructokinase gene *PFKFB1* which plays a key regulatory role in the glycolytic pathway, as well as the enzymes acetyl-coenzyme A carboxylase A and B (*ACACA* and *ACACB* respectively), which trigger a committed step fatty acid synthesis, were markedly upregulated in differentiated fBAT, SAT, E3, NP88, and NP110 cells, but much less so in differentiated C4ELS5.1, C4ELS5.5, and C4ELSR2. As in the undifferentiated progenitor state, the E3 line clustered most closely to fBAT and SAT cells (i.e., expression of *CIDEA*), but this line had low to undetectable levels of *UCP1* following differentiation*.* Nevertheless, it showed abundant induction of *ADIPOQ* and *LIPASIN* and shared with fBAT and SAT cells the induced expression of the transcriptional activators *CEBPA*, *CIDEA*, *CIDEC*, and the lipid metabolizing genes *AACS* and *ELOVL6*. In contrast, the cell lines C4ELS5.1, C4ELS5.5, and C4ELSR2, which clustered separately, showed induced expression of *CITED1*, *PIB5PA*, *PNMT*, and to a limited extent *UCP1* but relatively low levels of *ADIPOQ* and *LIPASIN*. The C4ELS5.1, C4ELS5.5, and C4ELSR2 lines also differed from the other lines and fBAT and SAT cells in expressing relatively high levels of transcripts for the epithelial nonvoltage-gated, amiloride-sensitive, sodium channel *SCNN1A*, *PNMT*, *PIB5PA*, *COL9A1*, and *ALDH1A3.*

The NP88 and NP110 lines most closely matched the pattern of gene expression of the fBAT cells, although like the other hEP lines they lacked expression of *COX7A1*. NP88 and NP110 induced *CITED1*, *ADIPOQ*, *LIPASIN*, and *UCP1* similar to cultured fetal BAT-derived cells but differed from fBAT, SAT, and E3 cells in that they expressed low to undetectable levels of *CIDEA*, and induced relatively high levels of *ITIH1* and *KCNK12.* The skeletal muscle marker *MYF5* reportedly expressed in BAT progenitors was not observed in any of the lines tested (Additional file 2: Table S2).

### Morphological and protein markers of BA cells in differentiated hEP cells

**Fig. 3:**
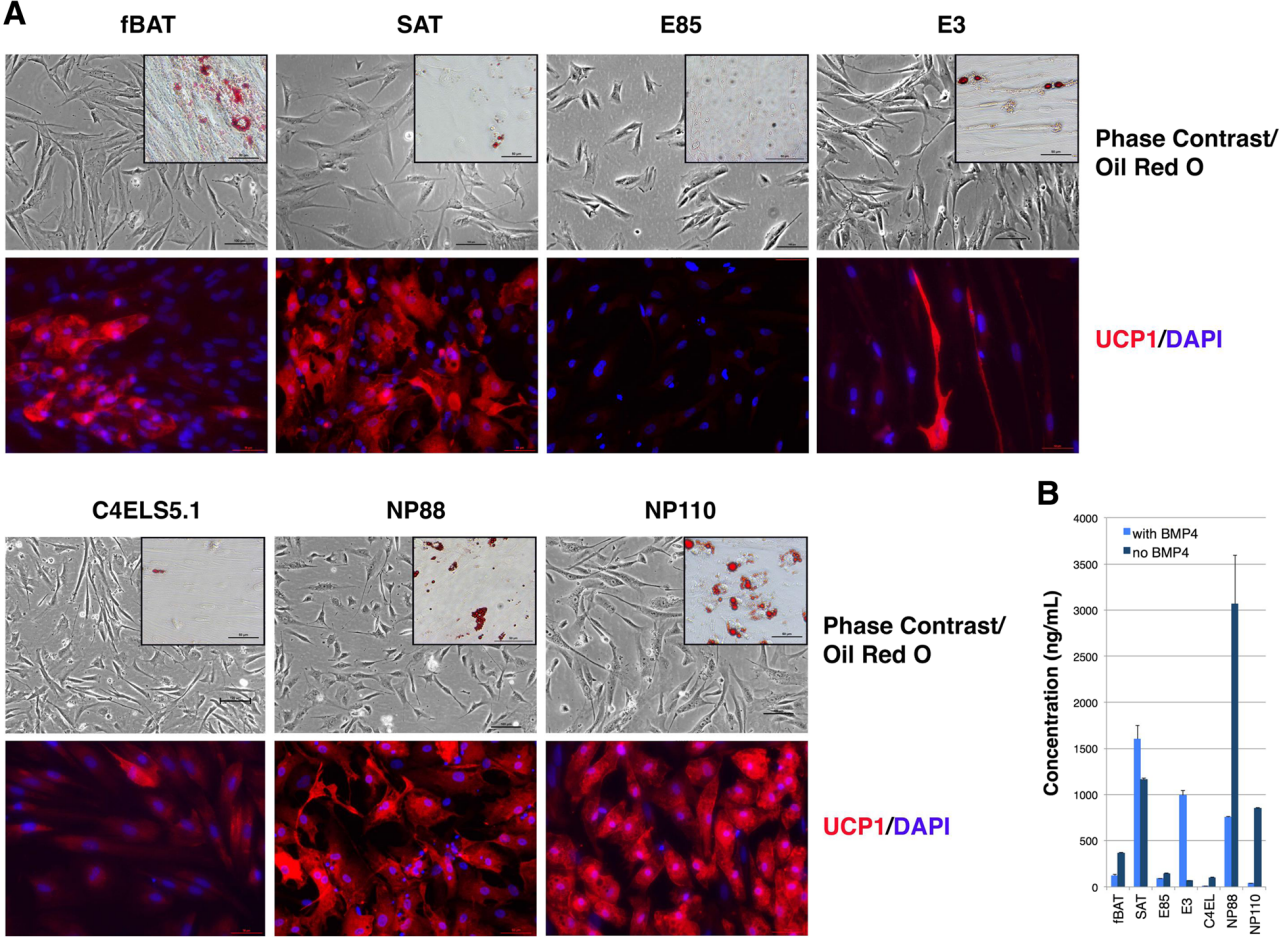
Comparative staining of cell types. **a** Top row shows phase contrast images of BAT- and WAT-derived preadipocyte cultures and the hEP cell lines E85, C4ELS5.1, C4ELSR2, E3, NP88, and NP110 in an undifferentiated state in log growth conditions overlain with inset showing Oil red-O staining after 14 days of adipogenic differentiation in HyStem-C with BMP4, rosiglitazone, T3, and CL316243 (BMP4, Rosi, T3, CL). Bottom row shows immunofluorescent detection of UCP1 in the respective cells cultured in the above-described adipogenic media using primary rabbit anti-human UCP1 polyclonal antibody and secondary Alexa Fluor 568 donkey anti-rabbit IgG antibody. Cell nuclei were stained using DAPI. **b** ELISA data quantifying adiponectin in lysates of cell lines in two differentiation conditions (BMP4, Rosi, T3, CL) and (Rosi, T3, CL). (Scale bar, 100 μm)

We undertook intact cell-based assays to confirm a subset of the markers on a protein level and determine the relative uniformity of the markers in each culture. The hEP lines as well as fBAT and SAT controls all displayed a similar mesenchymal/fibroblast-like morphology in log growth subconfluent culture prior to differentiation (Fig. 3a). After 14 days in HyStem-4D bead arrays supplemented with (Rosi, T3, CL), abundant multilocular lipid droplets accumulated in NP88 and NP110 as well as the control fBAT and SAT cells. Less abundant droplets were seen in E3 and C4ELS5.1 lines. In contrast, no lipid droplets were seen in the non-adipogenic line, E85 (Additional file 3: Figure S1). Oil red-O staining was used to confirm the presence of lipid droplets (Fig. 3a). Strong expression of UCP1 protein was detected in the clonal lines NP88 and NP110 and in fBAT and SAT control cells, but to a lesser extent in C4ELS5.1 and E3 (Fig. 3a). No anti-UCP1 antibody staining was detected in the negative control line, E85 (Fig. 3a), and the control antibody showed no staining in hEP or fBAT/SAT cells under the same conditions (Additional file 3: Figure S1). Mitochondria counts were elevated in all adipogenic hEP lines compared to fBAT preadipocytes. The negative control cell line, E85, had no increase in mitochondria relative to fBAT cells. The highest increase in mitochondria relative to fBAT was observed in differentiated NP110 cells (BMP4, Rosi, T3, CL) (Additional file 4: Figure S2, Additional file 5: Figure S3 and Additional file 6: Table S3).

**Fig. 4:**
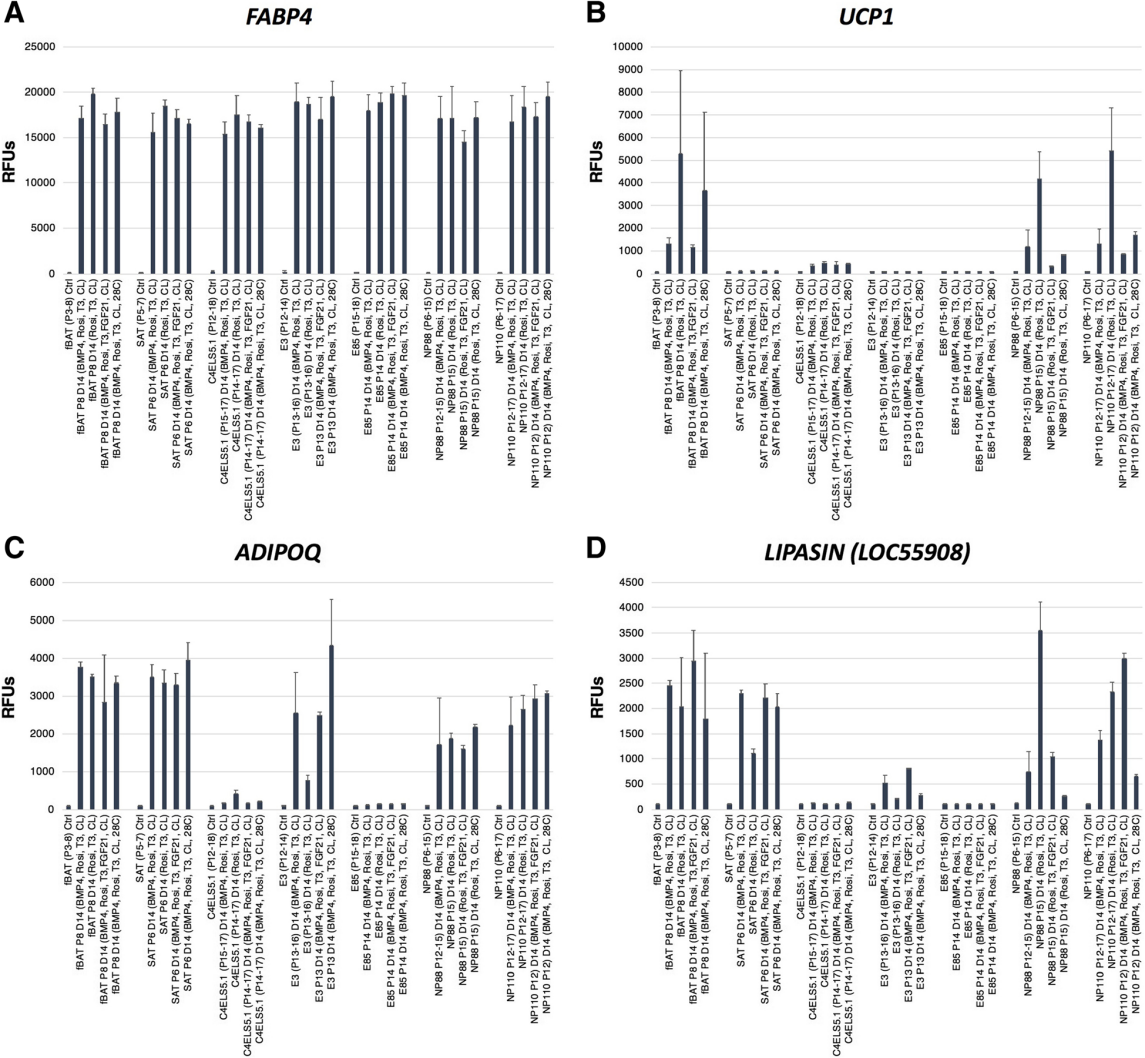
Relative levels of adipocyte gene expression in diverse differentiation conditions. **a** *FABP4*, **b** *UCP1*, **c** *ADIPOQ*, and **d** *LIPASIN* expression in the undifferentiated, 5-day quiescent cell clones and after 14 days of differentiation in HyStem-C with BMP4, rosiglitazone, T3, and CL316243 (BMP4, Rosi, T3, CL), HyStem-C with rosiglitazone, T3, and CL316243 (Rosi, T3, CL), HyStem-C with BMP4, rosiglitazone, T3, FGF21, and CL316243 (B4, Rosi, T, FGF21, CL), or HyStem-C with BMP4, rosiglitazone, T3, CL316243, and cultured at 28 °C. (BMP4, Rosi, T3, CL, 28C). (Error bars represent standard deviation)

Since adiponectin appears to play an important endocrine role in both WAT and BAT tissue, we chose it as an additional marker to confirm on a protein level. An ELISA-based assay for levels of adiponectin in whole cell lysates from cells differentiated in the (BMP4, Rosi, T3, CL) condition showed relatively high levels of the adipokine in fBAT, SAT, NP88, and E3 lysates. In contrast, we detected a very low level of protein in E85 and C4ELS5.1 extracts, which was consistent with the relatively low levels of *ADIPOQ* transcript in these lines (Figs. 3b and 4c). The omission of BMP4 from the differentiation media decreased adiponectin levels in the line E3 while increasing the levels in NP88 and NP110 (Fig. 3b).

### Optimization of BA progenitor cell line differentiation conditions

In addition to PPARG agonists such as rosiglitazone, low temperature [22], FGF21 [23], and diverse TGFβ family members [24, 25] have been reported to directly upregulating *UCP1* expression in BAT progenitors. To examine the relative effects of a subset of these alternative conditions, each cell type was cultured in the progenitor state synchronized 5 days in quiescence (Ctrl) or in one of four different differentiation conditions: (1) 14 days in HyStem-4D bead arrays supplemented with differentiation mix (BMP4, Rosi, T3, CL); (2) same as condition 1, but without BMP4 (Rosi, T3, CL); (3) same as condition 2, but with 50 ng/ml FGF21 for the last 2 days (Rosi, T3, FGF21, CL); and (4) same as condition 2, but incubated at 28 °C for the last 4 h (Rosi, T3, CL, 28 °C). Illumina bead array data was analyzed to determine levels of transcripts for *FABP4*, *UCP1*, *ADIPOQ*, and *LIPASIN*. As shown in Fig. 4, one or both of the NP88 and NP110 lines expressed relatively high levels of transcript for *UCP1* and *LIPASIN* compared to fBAT cells or the other hEP lines. *FABP4* was consistently expressed in all the lines. In the case of *UCP1* expression, there appeared to be a trend toward higher levels of expression in medium lacking BMP4.

**Fig. 5:**
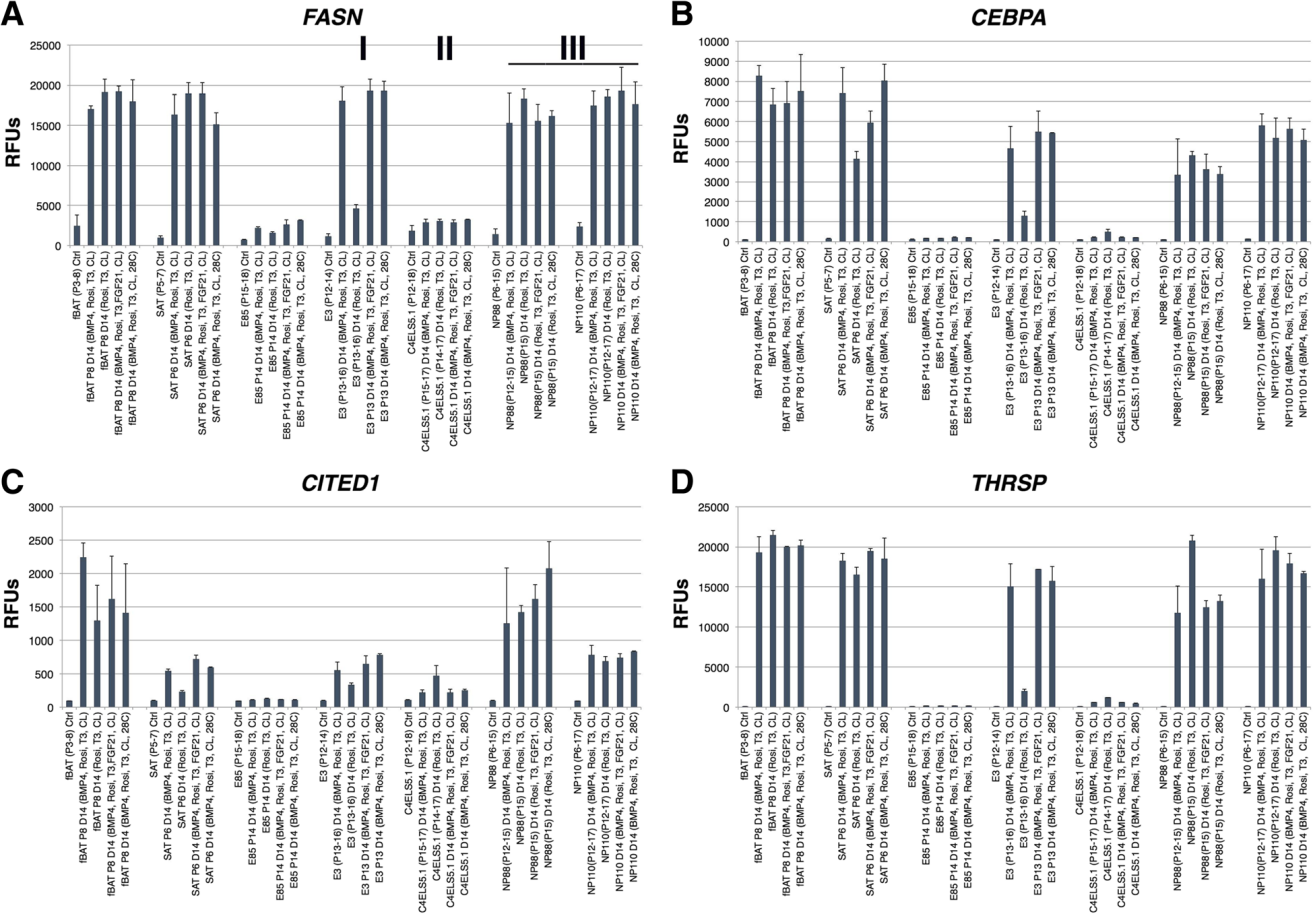
Comparative microarray-based adipogenic gene expression analysis of cell lines in four differentiation conditions. Data are displayed as mean values of two or more biological replicates generated on Illumina gene expression bead arrays for **a** *FASN*, **b** *CEBPA*, **c** *CITED1*, and **d** *THRSP*. Roman numerals designate class of clonal adipose progenitors. (RFU values < 130 considered background signal). (Error bars represent standard deviation)

Transcriptomic analysis by Illumina bead arrays was performed (Additional file 7: Table S4), and select adipogenic markers are shown in Fig. 5. The adipogenic markers *FASN*, *CEBPA*, *CITED1*, and *THRSP* showed differences among the lines. The non-adipogenic negative control progenitor line, E85, shows no induction of most of the adipose markers. The lines E3, NP88, and NP110 had levels of these and other adipocyte markers comparable to the adult-derived fBAT and SAT preadipocyte controls (Additional file 7: Table S4). The highest levels of the glucose transporter *SLC2A5* were seen in the differentiated clonal lines NP88 and NP110 and the control fBAT and SAT cells. It has been reported that BAT cells originate from progenitors of skeletal muscle and under the influence of *PDRM16* commit to a BAT cell fate [26]. Low, but detectable levels of *PDRM16* were observed in the lines C4ELS5.1, NP88, and NP110, but not in the other lines tested (Additional file 7: Table S4). The gene *DIO2* commonly used as a BAT marker [27] was expressed at relatively high levels in the non-adipogenic control lines E85 and C4ELS5.1, but at much lower levels in the other cells tested (Additional file 7: Table S4).

**Fig. 6:**
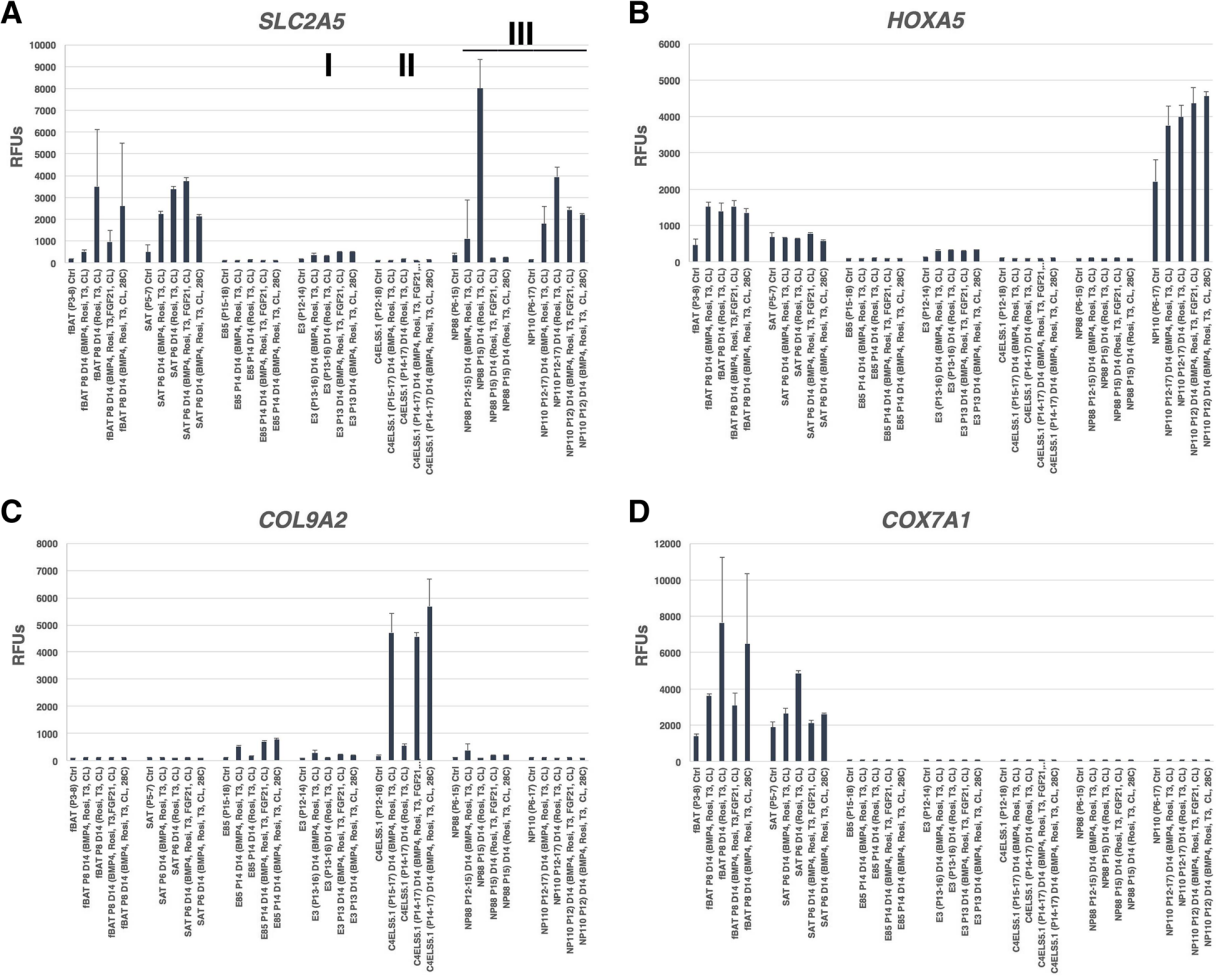
Differentially expressed genes in cell lines in four differentiation conditions. Data are displayed as mean values of two or more biological replicates generated on Illumina gene expression bead arrays for **a** *SLC2A5*, **b** *PRDM16*, **c** *HOXA5*, and **d** *COX7A1*. Roman numerals designate class of clonal adipose progenitors. (RFU values < 130 considered background signal). (Error bars represent standard deviation)

The conditions (BMP4, Rosi, T3, CL) and (Rosi, T3, CL) were used to test the relative influence of BMP4 on induction of adipocyte markers. TGFβ family members such as BMP4 are reported to induce browning [24]. As shown in Figs. 5 and 6 and Additional file 7: Table S4, the presence of BMP4 tended to increase adipocyte markers such as *CEBPA*, *THRSP*, *CITED1*, *MRAP*, and *FASN* in the line E3, but strongly induced the stromal tissue marker *COL9A2* in the lines C4ELS5.1 and *CRABP1* in the lines C4ELS5.1 and NP88. We also tested other conditions reported in the literature to induce browning such as the addition of FGF21 (Rosi, T3, FGF21, CL) and culturing for the final 4 h at 28 °C (Rosi, T3, CL, 28 °C), the latter condition reported to directly induce *UCP1* in adipocytes independent of adrenergic stimulation [22]. No clear reproducible trend toward increased *UCP1* or other BAT markers using either FGF21 or 28 °C incubation was detected. The use of other TGFβ family members BMP8B and GDF5 which are reported to induce *UCP1* did not induce BAT markers in our hands, although BMP7 had a modest effect compared to BMP4 (data not shown). Therefore, the optimal condition tested that resulted in the strongest induction of *UCP1* and *LIPASIN* expression (Fig. 4) and minimal induction of stromal differentiation markers such as *COL9A2* was Rosi, T3, CL.

It has been reported that thermogenic activation of BAT cells results in increased expression of *FGF21* [28]. As shown in Additional file 8: Figure S4 and Additional file 7: Table S4, a modest induction of *FGF21* was observed during the differentiation of NP110, less so in NP88, but no induction was detectable using Illumina arrays in the other cell types including fBAT cells.

Anatomically diverse BAT depots have been reported to show site-specific markers. In a study comparing markers in progressively deeper layers of fat in the neck, SAT cells shows the highest levels of leptin *(LEP)* transcripts, while deeper layers express *LHX8* and *UCP1* [27]. As shown in Additional file 8: Figure S4, SAT cells showed the highest relative levels of *LEP*, while only the non-adipogenic control E85 showed the mandibular and upper neck marker *LHX8* [29]. In another more precise study, *CD29* (also known as integrin, beta 1, *ITGB1*) and *ITGB10* were reported to be surface markers for clonal, *TERT*-immortalized, adult-derived BAT progenitors from diverse anatomical sites [30]. However, in our analysis of relative transcript levels by Illumina arrays, *ITGB1* appeared to be expressed in all cell types and conditions, with relatively high levels in the fBAT-derived preadipocytes, but relatively low levels in NP88 and NP110 progenitors (Additional file 7: Table S4). *ITGB1* was expressed in many other somatic cell types including CNS glial cells, diverse types of smooth and skeletal muscle cells, bone marrow MSCs, as well as others (data not shown). The marker *ITGB10* was expressed in relatively high levels in the progenitor cell lines NP88 and NP110, but was expressed in relatively low levels in fBAT preadipocytes. While not a cell surface antigen, a lack of *TSPO* expression, a drug target for diabetes and obesity [31], was observed to uniquely characterize undifferentiated NP88 and NP110 progenitors (Additional file 8: Figure S4 and Additional file 7: Table S4).

We previously reported that expression of the gene *COX7A1* provided a useful marker of the embryonic-fetal transition (embryonic stem cells, embryonic progenitors, and even differentiated embryonic cells lacking expression of the gene, with expression beginning in cells at the transition to fetal development) [19]. As shown in Fig. 6d, the progenitor lines do not express *COX7A1* in the control or differentiated state while fetal and adult-derived fBAT and SAT do express the gene consistent with their post-embryonic origin.

### Comparative metabolism in NP88 vs. fBAT cells

**Fig. 7:**
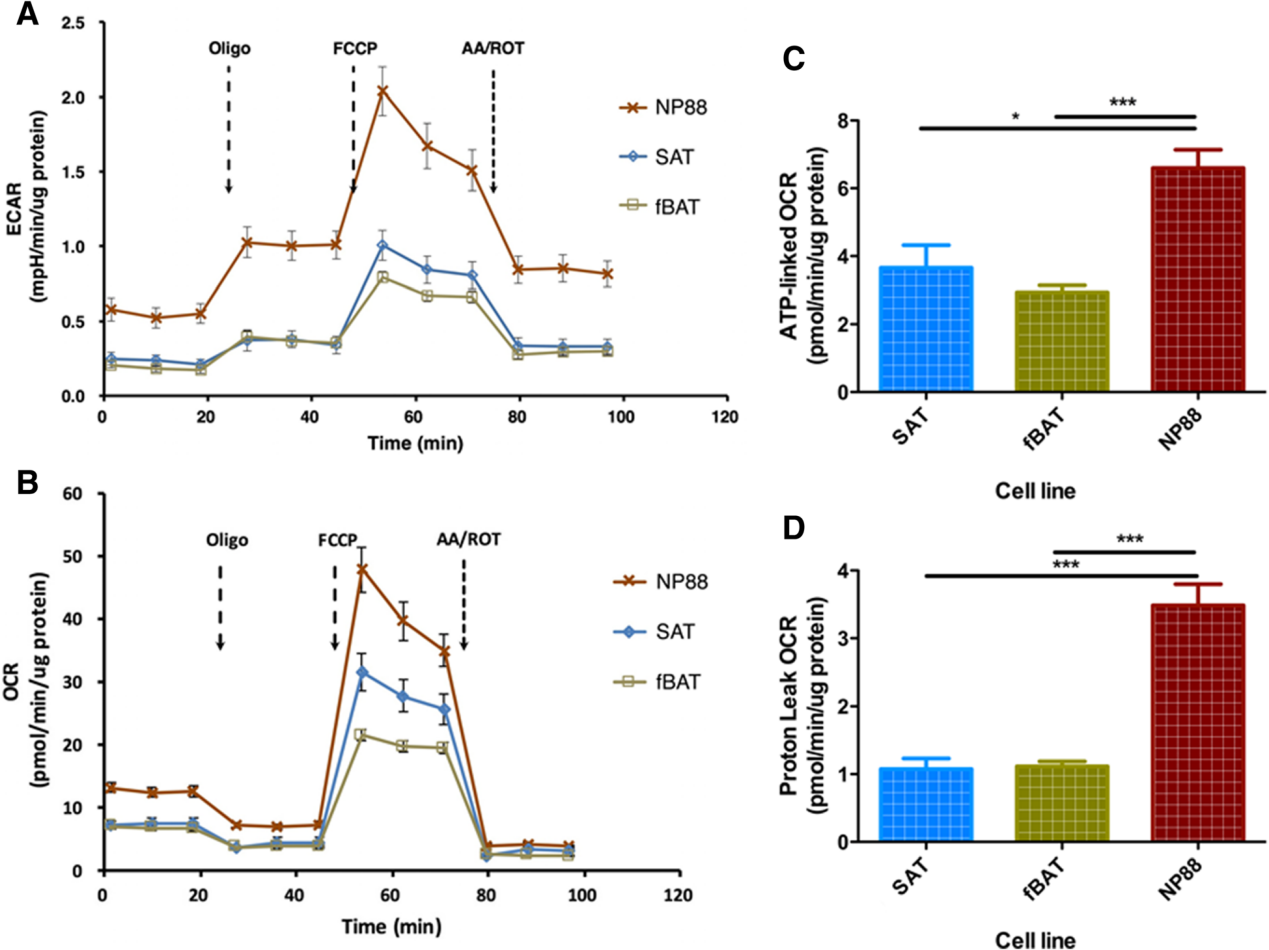
Extracellular flux of differentiated lines. NP88, fetal brown preadipocytes (fBAT), and subcutaneous preadipocytes (SAT) cells were differentiated in the presence of BMP4, rosiglitazone, and T_3_ for 14 days. Extracellular acidification rate (ECAR) and oxygen consumption rate (OCR) were measured in real time followed by the injection of mitochondrial respiration inhibitors using Seahorse XF24 Extracellular Flux Analyzer (**a** and **b**). ATP-linked OCR (**c**) and proton leak OCR (**d**) were calculated and plotted following Seahorse XF user guide. Data represent mean ± SEM, *n* = 9~16; *P < 0.05***P < 0.001 Student’s *t* test

The embryonic phenotype of NP88 and NP110 cells as evidenced by the absence of *COX7A1* expression could reasonably be expected to alter metabolism based on the role of the expressed protein, a subunit of mitochondrial complex IV, in OXPHOS supercomplex assembly [32]. This could be particularly relevant for the embryonic brown fat progenitors that we describe here given the relatively high levels of *COX7A1* expression in BAT compared to other tissues [33]. As shown in Fig. 7a, differentiated NP88 cells (1uM rosiglitazone, 2.0 nM T3, and ITS) had higher levels of basal extracellular acidification compared to differentiated fBAT and SAT cells, consistent with the increased glycolytic capacity of embryonic (*COX7A1-*) cells [19]. The differentiated embryonic NP88 cells also had significantly increased basal oxygen consumption (ATP-linked OCR) as well as more robust increase in oxygen consumption to maximal respiration upon uncoupling with the protonophore FCCP (Fig. 7b, c) compared to differentiated fBAT or WAT. Proton leak OCR was also significantly increased in differentiated NP88 cells compared to differentiated fBAT and SAT (Fig. 7d).

We also sought to understand differences in the metabolome of the embryonic BA progenitors versus fetal/adult progenitors. The metabolic signature of NP88 and fBAT in the undifferentiated vs. differentiated state were analyzed on RP and HILIC/MS/MS platforms.

**Fig. 8:**
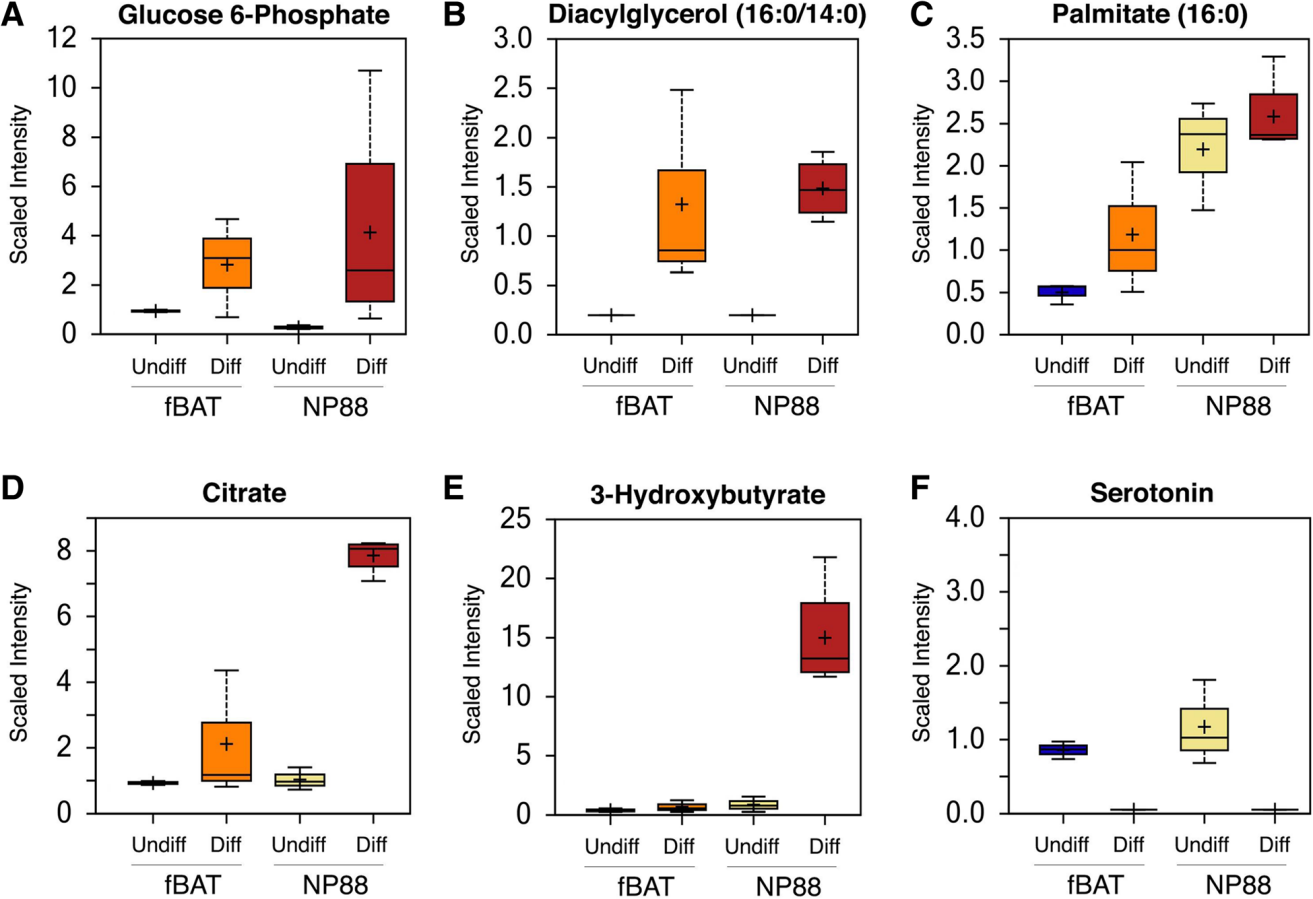
Relative levels of cell metabolites were obtained using non-targeted liquid chromatography–tandem mass spectroscopy. Box plots for cell metabolites **a** glucose-6 phosphate, **b** diacylglycerol, **c** palmitate, **d** citrate, **e** 3-hydroxybutyrate, and **f** serotonin are shown for undifferentiated and differentiated (in serum-free medium containing rosiglitazone, T_3_, and ITS for 14 days) fBAT and NP88. The plus sign represents the mean value, and the line in the box is the median. The uppermost and lowermost bar represent the minimum and maximum of the distribution while the upper and lower side of the box is the upper and lower quartile

The data showed that each cell line demonstrated a unique metabolic signature. Almost 50% of the metabolites were found to be significantly different between the adult fBAT and embryonic NP88 cells (Additional file 9: Table S6). Lipid metabolites were among those that were most different between adult and embryonic cell lines. As shown in Fig. 8a-d and Additional file 9: Table S6, glucose, triglyceride, and citric acid cycle metabolites were higher in differentiated fBAT and NP88 cells compared to their preadipocyte progenitors. In addition, differentiated fBAT and NP88 cells showed lower levels of serotonin (Fig. 8f), reported to inhibit BAT cell function while undifferentiated fBAT and NP88 had relatively higher levels. Lastly, embryonic NP88 cells showed markedly higher levels of the ketone body metabolite 3-hydroxybutyrate which accompanies calorie-restrictive ketogenic diets and has immunosuppressive activity (Fig. 8e), consistent with the absence of *COX7A1* expression.

### Scalability and re-derivation of NP110 cells

The purity of clonal embryonic progenitor cell lines such as NP88 or NP110 could facilitate the manufacture of clinical-grade BAT cells if the progenitors can be propagated on an industrial scale. We therefore examined the stability of *UCP1* expression in NP110 following serial passaging. As shown in Additional file 10: Figure S5, *DLK1* that was abundantly expressed in both NP88 and NP110 in the undifferentiated progenitor state (Fig. 2 and Additional file 2: Table S2) was maintained in NP110 through P21, but was lost by P32 (Additional file 10: Figure S5). *CITED1* expression also decreased to undetectable levels by P32. In contrast, the stromal marker *COL11A1* that was not expressed in NP110 in the progenitor state showed progressively increased expression as the cells were propagated. As we have already shown, the marker *FABP4* was not specific to definitive adipogenesis and was robustly induced in the line at all passages studied. Importantly, the ability to induce *UCP1* expression was maintained at least to P17 but lost by P27. The clonal expansion of cells from a single cell to a confluent well that can be serially propagated to P17 would correspond to a theoretical expansion potential of 7 × 10^9^ cells. While the required dosage for therapeutic application would vary, it is likely that this number of cells would correspond to only a relatively small number of doses compared to the potential patient population. We therefore examined whether the heterogeneous culture of cells from which the line was cloned designated a candidate culture [14] could yield additional lines with similar fate potential. This would potentially allow the production of many doses from a single candidate culture and therefore provide a point of scale out in addition to scale up.

We isolated nine additional clonal lines from the original cryopreserved heterogeneous candidate culture that yielded NP110. As shown in Additional file 11: Figure S6 and Additional file 12: Table S5, 6/9 newly isolated clones yielded lines capable of a combination of *UCP1*, *ADIPOQ*, *LIPASIN*, *and CITED1* expression upon differentiation. In addition, 1/9 clones showed similar site-specific markers as NP110 such as *HOXA5*. Other lines showed expression of *DLX5* in either the undifferentiated or differentiated state, perhaps evidence of an additional osteogenic potential for those lines.

## Discussion

We previously reported the clonal derivation and characterization of hundreds of hEP cell lines from hES cells and demonstrated > 140 distinct cell types by NMF [14]. These hEP lines can be extensively scaled in vitro [29] and further screened for cell fate identity by differentiating in micromass or HyStem-4D bead arrays [18] to determine their response to specific differentiation signals [16, 34]. Since HyStem-C, which is used to produce HyStem-4D bead arrays, is produced under cGMP and is currently being utilized in a clinical trial to augment cell-assisted lipotransfer for the treatment of HIV-related lipoatrophy (*Renevia*), we began a screen of randomly selected hEP cell lines in HyStem-4D bead arrays in adipogenic conditions to determine whether a subset of the lines was capable of differentiation into diverse adipogenic fates.

While certain genes such as *FABP4* and *CD36* are commonly used as markers of adipocyte differentiation, we observed that the majority of embryonic progenitor cell lines tested were capable of markedly inducing these genes in HyStem-C adipogenic differentiation medium containing BMP4, rosiglitazone, triiodothyronine (T3), and the β3 adrenergic receptor agonist CL316243 even though they lacked observable lipid accumulation after 14 days of differentiation. However, a smaller subset of lines showed the capacity to upregulate combinations of *UCP1*, *ADIPOQ*, *LIPASIN*, or *CITED1* which we interpreted as definitive markers of adipogenesis since these lines had observable Oil red-O stained lipid accumulation following differentiation*.* An example of a line that expressed *FABP4* and *CD36* but not the other four adipogenic markers following induction is the hEP line E85. This cell line was chosen as a negative control line for adipogenesis due to its lack of lipid accumulation under differentiation conditions.

All the clonal lines displayed a mesenchymal/fibroblast-like morphology similar to fBAT and WAT preadipocytes when cultured in standard propagation conditions. When induced to differentiate in adipogenic conditions, robust multilocular lipid vesicles were observed in lines expressing *CITED1*. While fully mature BAT and WAT cells have been reported to contain multilocular and unilocular lipid respectively, immature WAT cells have also been reported to be multilocular [35] perhaps consistent with the relative immaturity of the cells differentiated herein.

The subset of lines chosen for further study fell into three classes: Class I represented by the cell line E3 showed a potential to induce *CITED1*, *ADIPOQ*, and *LIPASIN* expression in response to adipocyte differentiation conditions, but little or undetectable *UCP1.* Because this line also expressed *HOX* gene markers consistent with the upper limb girdle, these cells may therefore represent the embryonic counterpart of SAT cells. Further study of the line in comparison with diverse types of mature adipocytes would need to be performed to draw definitive conclusions.

Class II cells represented by C4ELS5.1 expressed relatively low levels of *UCP1* and low to undetectable levels of *ADIPOQ* and *LIPASIN.* The expression of the homeobox gene *PITX1* in two of the other lines in this class (C4ELS5.5 and C4ELSR2) in the absence of distal (leg) *HOX* gene expression suggests C4ELS5.1 may correspond to the neck region of embryonic development and perhaps represent a type of BAT distinguishable from BAT isolated from the trunk.

Class III lines, NP88 and NP110, in contrast, strongly induced *ADIPOQ*, *LIPASIN*, and *UCP1* similar to that observed in differentiating fBAT and SAT cells. However, unlike fBAT, SAT, and the E3 cells, the class III embryonic progenitors expressed low to undetectable levels of *CIDEA* transcript. Since *CIDEA*-null mice have been reported to have a lean phenotype and resistance to diabetes [36], class III lines may represent definitive BA progenitors with certain advantages over fetal or adult-derived BAT cells for potential use in transplantation therapy. In addition, the class III line, NP88, when differentiated was shown to contain markedly elevated 3-hydroxybutyrate compared to differentiated fBAT cells. Since 3-hydroxybutyrate has been implicated in mediating beneficial effects of dietary restriction including inhibition of inflammation, this characteristic of embryonic BAT as opposed to adult-derived BAT may also provide unique advantages in research and therapy.

Differences in site-specific homeobox gene expression were also observed in the hEP cell clonal lines. The most rostral expression of *HOXA5* is generally observed at about the region of the shoulder girdle; therefore, NP110 may represent brown adipocyte progenitors from approximately the region of interscapular BAT cells. In contrast, the lack of distal *HOX* expression in the line NP88 may reflect an anatomical assignment of the neck BAT to this line. Additional studies will be required to more precisely correlate these in vitro differentiated adipocyte progenitors with the corresponding cells in vivo.

A comparison of the gene expression markers *UCP1*, *ADIPOQ*, and *LIPASIN* in the lines under the diverse differentiation conditions studied suggests that the lines NP88 and NP110 were optimally differentiated in HyStem-C supplemented with 1 μM rosiglitazone, 2 nM triiodothyronine (T3), and for the final 4 hours 10 μM of the β3 adrenergic receptor agonist CL316243, but in the absence of BMP4. Shifting the cells to 28 °C did not appear to have an effect on differentiated gene expression.

It has been reported that activated brown adipocytes have the highest uptake of glucose in the human body. This is consistent with the relatively high levels of glucose, glucose 6-phosphate, and its metabolites in differentiated fBAT and class III cells. Therefore, understanding the regulatory mechanisms of this uptake could yield important new targets for therapy. It is reported that the induction of *GLUT1* (*SLC2A1*) and *GLUT4* (*SLC2A4*) glucose transporters play an important role in regulating glucose uptake in brown adipocytes [37]. However, we observed *SLC2A1* to be downregulated during differentiation in most cell types and little evidence of upregulation of *SLC2A4* (Additional file 7: Table S4). However, relatively high levels of induction of the glucose transporter *(GLUT5) SLC2A5* were seen in the differentiated clonal lines NP88 and NP110 and the control fBAT and SAT cells. As a result, a study of the molecular regulation of SLC2A5 expression may provide useful information in the search for novel drug targets and it would be informative to compare pluripotent stem cell-derived BAT cells in a physiologically compatible matrix such as HyStem-C in animal models of type II diabetes to determine whether the transplanted tissue could help normalize circulating glucose levels.

*DLK1* appears to play an important role in muscle development [38] as well as being an adipogenic regulator [20]. Most studies implicate *DLK1* in inhibiting preadipocyte proliferation and differentiation. The relatively high levels of expression of *DLK1* in NP88 and NP110 in the progenitor state do not necessarily contradict this assertion as there are different splice variants that can alter the propensity of the protein to be cleaved and solubilized and a study of such variants was not performed in this report.

Reports relating to BAT depots residing in diverse anatomical locations such as perivascular, paravertebral, or renal sites suggest that there may be subtle differences in function depending on location within the body [39]. The expression of *HOXA5* in NP110, but not in C4ELS5.1 or NP88, suggests clonal BAT embryonic progenitors with site specificity can be isolated similar to that reported previously with diverse osteochondral progenitors [17]. Since altered expression of *HOXA5* is reported to lead to homeotic transformations in the C3-T2 axial skeleton [40], it is possible NP110 corresponds to BAT at this anatomical site, although further studies to confirm this identification are required. Comparative studies could be facilitated by detailed annotated embryonic and adult cell line transcriptomic databases such as that provided by LifeMap Discovery [41].

The absence of *COX7A1* expression in the hEP cell-derived BAT cells was striking. While both fetal-derived fBAT and adult-derived SAT cells expressed the gene in both the progenitor and differentiated states, none of the hEP cell-derived lines either in the progenitor or their differentiated counterparts expressed this marker of the embryonic-fetal transition. While commonly thought of as a muscle/BAT cell marker, *COX7A1* is widely expressed in numerous tissues following the embryonic-fetal transition [19]. It seems reasonable to conclude that this evidence of the embryonic developmental stage of the hEP cell-derived BAT cells does not suggest incomplete differentiation since development of most tissues is essentially complete at the close of embryonic development. In addition, *Cox7a1* is not essential for functional BAT development because *COX7A1* knockout mice appear to have normal BAT activity [42].

Class III hEP-derived BAT cells showed evidence for the synthesis of adipogenic mediators such as lipasin, adiponectin, 3-hydroxybutyrate and potentially for metabolizing serotonin. Inhibiting peripheral serotonin levels has been implicated in reducing obesity through the induction of BAT cell thermogenesis [43]. Therefore, the transplantation of hEP-derived BAT cells may provide an endocrine effect in addition to the potential role of the tissue in metabolizing glucose and triglycerides. This endocrine effect may be enhanced when the cells are engrafted in a biocompatible extracellular matrix such as HyStem-C (marketed for human use as Renevia) to promote survival in vivo.

## Conclusions

Our transcript and metabolic analysis of the adipogenic potential of hES cell-derived clonal hEP cell lines provides evidence that self-renewing progenitors that are intermediate between pluripotency and differentiated WAT or BAT cells can be propagated in vitro in a manner similar to adult-derived preadipocytes. The clonal purity and scalability of hEP cell lines could potentially facilitate reproducibility when comparing diverse protocols on the same lines in different laboratories compared to using various primary cells of limited expansion capability. In addition, there is the potential to derive similar clonal BA progenitor lines from iPSC lines from donors with inherited metabolic disorders or from genetically modified hPS cell lines that could be valuable for disease modeling, drug screening, and therapeutic applications. The class III lines described here appear similar to fBAT but with unique qualities that may be advantageous for translational studies aimed at cell transplantation approaches to treating obesity and type II diabetes.

## Materials and methods

### Cell lines and growth factors

Cells lines were derived as previously described [14] in their respective propagation media: NP110 and NP88 were cultured in PromoCell Smooth Muscle Medium 2 with supplements (PromoCell, Cat. # C-22062); C4ELS5.1 and C4ELSR2 were cultured in EpiLife Medium (Life Technologies, phenol red free, Ca free Cat. # M-EPIcf/PRF-500), with LSGS (low serum growth supplement (Life Tech S-003-10)); E3 was cultured in DMEM high glucose with 20% FBS; E85 was cultured in DMEM high glucose with 10% FBS. All media were supplemented with 2 mM glutamax and 1× pen/strep. Fetal brown preadipocytes and subcutaneous preadipocytes were cultured in Subcutaneous Preadipocyte Medium (ZenBio, cat# PM-1). E3 and E85 were derived from the human embryonic stem cell line ACT03 (MA03). The lines C4ELS5.1, C4ELS5.5, and C4ELSR2 were derived from the human embryonic stem cell line H9 (National Institutes of Health-registered as WA09). The lines NP88 and NP110 were derived from the human embryonic stem cell line hES3. The hEP cell lines were propagated in the progenitor state (undifferentiated) by routine passaging prior to the cells becoming confluent at 37 °C in a humidified atmosphere of 10% CO_2_ and 5% O_2_ on gelatinized culture vessels in media per manufacturer’s instructions (BioTime, Alameda, CA, USA). The relatively high concentration of CO_2_ provides physiological pH in relatively low concentrations of O_2_. The hEP cell lines were serially passaged as previously described, while confluence was carefully prevented for more than 2 days to prevent differentiation while in the progenitor state. Gene expression in the relatively undifferentiated progenitor (or control) state utilized cells induced into a relative state of quiescence by changing the media in a just-confluent culture for media supplemented with 10% of the normal sera and growth factor supplements. For differentiation experiments. BMP4 was obtained from Humanzyme (Chicago, IL, USA), rosiglitazone from Torcis (Bristol, UK), T3 from Sigma (Saint Louis, MO), CL316243 from Torcis (Bristol, UK), and FGF21 was obtained from Peprotech (Rocky Hill, NJ).

### Differentiation in HyStem-4D bead arrays

HyStem-C (BioTime, Alameda, CA, USA) was prepared according to the manufacturer’s instructions. The Hystem C kit consists of three reagents that need to be reconstituted in degassed deionized water. Briefly, the HyStem component (thiol-modified hyaluronan, 10 mg) was dissolved in 1.0 mL to prepare a 1% *w*/*v* solution, the Gelin-S component (thiol modified gelatin, 10 mg) was also dissolved in 1 mL water to prepare a 1% w/v solution, and PEGDA (PEG diacrylate, 10 mg) crosslinker was dissolved in 0.5 mL to prepare a 2% *w*/*v* solution. Then, 1 mL HyStem was mixed with 1 mL Gelin-S just before use. Pelleted cells were resuspended in the HyStem:Gelin-S (1:1 *v*/*v*) mix, followed by the addition of crosslinker PEGDA, to yield a final cell suspension concentration of 2.0 × 10^7^ cells/mL. The cell suspension was aliquoted at 25 μL/bead into 6-well plates (Corning 3516) after partial gelation (typically five beads per well). Differentiation medium was added to each well following complete gelation (20–40 min). Plates were placed in a humidified incubator at 37 °C, ambient O_2_, 10% CO_2_, and the cells were fed fresh differentiation medium three times weekly. The hydrogel constructs are either fixed for immunohistochemical analysis, or lysed using RLT (Qiagen, Valencia CA), for total RNA to analyze transcript expression (using whole genome microarray), at the desired time points.

### Immunocytochemical fluorescence detection of UCP1

Embryonic progenitor and adult-derived cells, exposed to differentiation medium for 14 days at confluence, were washed once with PBS and fixed in 4% paraformaldehyde for 30–60 min at room temperature (RT). Fixed cells were washed three times with PBS, permeabilized and blocked by incubation in blocking buffer (5% normal donkey serum, 1% BSA and 0.1% Triton X-100 in PBS) for 1 h at RT. The cells were then incubated overnight at 4 °C with primary rabbit anti-human UCP1 polyclonal antibody (Thermo Sci. PA1-24894) at a dilution of 1:500 in 5% normal donkey serum, 0.5% BSA and 0.05% Triton X-100 in PBS. Then, the cells were washed four times with PBS plus 0.05% Triton X-100 (PBS-Triton) and incubated for 1 h at RT with Alexa Fluor 568 donkey anti-rabbit IgG antibody (Invitrogen, A10042) at a 1:500 dilution in PBS-Triton. Isotype controls were stained under identical conditions except that total rabbit IgG (Life Technologies, 10500C) was used as primary antibody (at the same concentration with UCP1 Ab, 1.34 μg/ml). Cells were counterstained with DAPI at 0.1 ng/mL for 10 min at RT and *imaged* on a *Nikon Eclipse TE2000*-*U* inverted microscope.

### Adiponectin quantitation

#### Sample preparation

The various cell lines were differentiated at confluence for 14 days then lysed with 0.6 ml of RIPA lysis buffer (Life Technologies Cat# 89900) plus proteinase inhibitor cocktail (Sigma Cat# P2714). The samples were stored at − 80 °C until analysis. They were later thawed and centrifuged at 20,000 g for 10mins at 4 °C. The supernatants were transferred to fresh Eppendorf tubes for BCA assay and ELISA. Remaining aliquots of samples were stored at − 80 °C for future use.

#### BCA assay

Total protein concentration was measured using the Pierce BCA Protein Assay Kit following the manufacturer’s instructions (ThermoFisher Scientific Cat# 23227). OD at 562 was measured using a BioTek Synergy HT microplate hybrid reader.

#### ELISA assay

ELISA was performed to measure adiponectin levels in the differentiated cell lysates using kits from Abcam (Cat# ab99968) following the manufacturer’s instructions. Samples and standards were incubated at 4 °C overnight; then, the OD at 450 nm was measured using a BioTek Synergy HT microplate hybrid reader. Values for each sample were calculated from the standard curve, and the average of duplicates was normalized to total protein concentration of the corresponding sample.

### Mitochondrial analysis

All progenitor and adult-derived cell lines were grown to confluence in 96-well plates in propagation media described above prior to addition of differentiation media. Basal differentiation media was prepared using DMEM (CellGro Cat. No. 15-013-CV, or PromoCell, Heidelberg Germany C-71219), high glucose, 1 mM Pyruvate (Gibco Cat. 11360), 100 U/mL:100 μg/mL Pen:Strep (Gibco Cat. No. 504284), 2 mM Glutamax (Gibco Cat. No. 35050), 0.1 μM Dexamethasone (Sigma, St. Louis, MO, Cat. No. D1756-100), 0.35 mM L-Proline (Sigma Cat. No. D49752), 0.17 mM 2-phospho-L-Ascorbic Acid (Sigma, Cat. No. 49792, Fluka), ITS Premix (BD, Franklin Lakes, NJ, sterile Cat. No. 47743-628) final concentration 6.25 μg/ml insulin, 6.25 μg/ml transferrin, 6.25 ng/ml selenious acid, 1.25 mg/ml serum albumin, and 5.35 μg/mL linoleic acid. Complete differentiation media consisted of basal medium with 10 ng/ml BMP4, 1 μM rosiglitazone, 2 nM T3 or basal medium with 1 μM rosiglitazone and 2 nM T3. Cells were differentiated for 14 days with fresh media changed every 2 days. Each condition was replicated in triplicate. On day 14, the cells were treated with 10 μM CL316243 4 h. The cells were then treated with 5 μM MitoTracker Deep Red FM (Thermo Scientific cat#M22426) and 1 μg/mL Hoechst 33342 (Thermo Scientific cat#62249) for 30 min. The cells were then fixed for 15 min using 4% PFA. Fixation was removed and cells were washed two times with PBS prior to high content image analysis using a ThermoFisher ArrayScan XTI. Twenty-five images from each well were collected. Cells were identified, and mitochondria content per cell was analyzed using ArrayScan Studio 2 analysis software. Error bars are ±SEM, and statistics were calculated using Student’s *t* test for each condition against basal control.

### Gene expression analysis

Total RNA was extracted from cells using Qiagen RNeasy mini kits according to instructions supplied by the manufacturer. RNA concentrations were measured using a Nanodrop spectrophotometer, and RNA integrity was determined by denaturing agarose gel electrophoresis or by an Agilent 2100 bioanalyzer. Whole-genome expression was obtained using Illumina Human HT-12 v4 BeadArrays. In preparation for Illumina BeadArrays, total RNA was linearly amplified and biotin-labeled using Illumina TotalPrep kits (Life Technologies, Temecula, CA, USA). The cRNA quality was measured using an Agilent 2100 Bioanalyzer before being hybridized to Illumina BeadChips, processed, and read by an iScan microarray scanner according to the manufacturer’s instructions (Illumina, San Diego, CA, USA). Values under 130 relative fluorescence units (RFUs) were considered as nonspecific background signal.

#### Data analysis

Analysis of microarray data was performed using the R lumi library [44]. Raw microarray data were normalized with the R beadarray library. Merging of data from different experiments and their subsequent quantile normalization was performed using functions combine and lumiN, respectively, of lumi library. Dendrograms were created by Hierarchical cluster analysis applying average agglomeration method. Heatmaps were created in GeneSpring suite.

### Metabolome analysis

Metabolome analysis (Metabolon, Inc., Durham, NC) was conducted on cell pellets and media collected from fBAT cells (P7) and NP88 cells (P16) in the undifferentiated state (preadipocyte) cultured 5 days in 0.5% FBS-containing DMEM medium, as well as the differentiated state (at confluence for 14 days (fed three times weekly) with serum-free basal medium and rosiglitazone 1 μM + T3 2 nM and supplemented with ITS Premix (BD, Franklin Lakes, NJ, sterile Cat. No. 47743-628) final concentration 6.25 μg/ml insulin, 6.25 μg/ml transferrin, 6.25 ng/ml selenous acid (ITS)). A non-targeted relative quantitative liquid chromatography–tandem mass spectrometry RP and HILIC)/MS/MS platforms were applied to identify structurally named and unknown molecules [45, 46]. All normalized relative ion counts were log transformed, and the remaining data were imputed with the minimum value on a per metabolite basis.

### Extracellular flux measurement

Oxygen consumption rate (OCR) and extracellular acidification rate (ECAR) were measured using the Seahorse XF24 Extracellular Flux Analyzer as described. NP88 and fetal brown preadipocytes (fBAT) and subcutaneous preadipocytes (SAT) cells grew to confluent in gelatin-coated V7 cell culture plates (Agilent, CA) and differentiated for 14 days in the presence of BMP4, Rosi, and T_3_ as previously described. Immediately before XF assay, cells were equilibrated with unbuffered XF assay medium supplemented with 17.5 mM glucose, 10 mM sodium pyruvate, and 2 mM glutamate and incubated in a 37 °C non-CO_2_ incubator for 1 h. Adrenergic stimulators (final concentration 10 μM) or mitochondrial respiration inhibitors were prepared in the identical assay medium and were injected from the reagent ports automatically to the wells at the time as indicated. The final concentrations of compounds: 1.5 μM Oligomycin, 1.5 μM FCCP, and 1 μM Rotenone-Antimycin A.

## Additional files

Additional file 1: **Table S1.** Illumina-based microarray data on expression levels of *FABP4*, *UCP1*, *ADIPOQ*, *CITED1*, *LIPASIN*, and *MYH3*, from control cells and 20 random hES cell-derived clonal embryonic progenitor cell lines differentiated for 14 days in (BMP4, Rosi, T3, CL) used in support of Fig. 1. Values less than 130 RFU were considered to be background signal. (XLSX 14 kb)

Additional file 2: **Table S2.** Illumina-based microarray data on fBAT and SAT cells and the hES cell-derived clonal embryonic progenitor cell lines E85, E3, C4ELS5.1, C4ELS5.5, C4ELSR2, NP88, and NP110 in progenitor state (Ctrl) or differentiated for 14 days in (BMP4, Rosi, T3, CL). Calculation of mean RFUs in control (CTRL) and differentiated conditions for each line used in support of Fig. 2a (sheet 2) and Fig. 2b (sheet 3) are shown. Values less than 130 RFU were considered to be background signal. (XLSX 11511 kb)

Additional file 3: **Figure S1.** Phase contrast photographs of the cell lines used after 14 days of differentiation in (BMP4, Rosi, T3, CL). Also shown are antibody controls to the primary rabbit anti-human UCP1 polyclonal antibody and secondary Alexa Fluor 568 donkey anti-rabbit IgG antibody. Cell nuclei were stained using DAPI. (JPG 42552 kb)

Additional file 4: **Figure S2.** Microscopic photographs of cells following mitochondrial staining in the progenitor state (Ctrl), in (BMP4, Rosi, T3, CL) and (Rosi, T3, CL) differentiation conditions. (JPG 8400 kb)

Additional file 5: **Figure S3.** Quantitation of mitochondria in the cells in the progenitor state (Ctrl), in (BMP4, Rosi, T3, CL) and (Rosi, T3, CL) differentiation conditions. (JPG 466 kb)

Additional file 6: **Table S3.** Mitochondria quantitation data used in support of Additional file 3: Figure S1 (sheet 1) and ELISA data used in support of Fig. 3b (sheet 2) are shown. (XLSX 16 kb)

Additional file 7: **Table S4.** Illumina-based microarray data is presented for normalized data on all gene expression (sheet 1), the calculation of mean values and standard deviation (sheet 2), the data used in support of Fig. 4 (sheet 3), the data used in support of Fig. 5 (sheet 4), the data used in support of Fig. 6 (sheet 5), the data used in support of Additional file 4: Figure S2 (sheet 6) and sugar transporter data (sheet 7). (XLSB 99804 kb)

Additional file 8: **Figure S4.** Illumina-based RFU values for the expression of *FGF21, LEP*, *LHX8*, *ITGA1*, *ITGA10*, and *TSPO* in the lines under four diverse differentiation conditions. (JPG 6489 kb)

Additional file 9: **Table S6.** A non-targeted relative quantitative liquid chromatography–tandem mass spectrometry RP and HILIC)/MS/MS platform was used to capture 624 cell metabolites and 406 metabolites in their respective media at time of harvest. Data is shown for fBAT and NP88 in undifferentiated and differentiated (in serum-free medium containing rosiglitazone, T_3,_ and ITS for 14 days) cells. Data for other progenitor and adult cell lines is also presented in this table. (XLSX 474 kb)

Additional file 10: **Figure S5.** Expression of select genes in the line NP110 at increasing passage numbers determined by Illumina Bead array. Cell line NP110 was cultured in D14 HyStem differentiation conditions with or without BMP4. Data are displayed as mean values (*n* = 3 ± standard deviation). (JPG 2816 kb)

Additional file 11: **Figure S6.** BAT differentiation potential of clonal lines that were re-derived from candidate culture used to derive NP88 and NP110. Data are displayed as mean values of two or more biological replicates generated on Illumina gene expression bead arrays for (A) *UCP1*, (B) *ADIPOQ*, (C) *LIPASIN*, (D) *CITED1*, (E) *HOXA5*, and (F) *DLX5*. (*) marks clones designated clonal BAT progenitors. (RFU values < 130 considered background signal). (Error bars represent standard deviation). (JPG 4167 kb)

Additional file 12: **Table S5.** Illumina-based microarray data is presented for the expression of Illumina-based microarray mean values and standard deviation for the genes *FABP4*, *UCP1*, *ADIPOQ*, *CITED1*, *LIPASIN*, *MYH3*, *HOXA5*, and *DLX5* in fBAT, SAT, NP88, NP110, and nine new clonal isolates in the progenitor (Ctrl) state and after 14 days of differentiation in (BMP4, Rosi, T3, CL) in an effort to re-derive clonal progenitors to BAT. (XLSX 13 kb)

## Abbreviations

BAT: Brown adipose tissue
dH20: Distilled water
fBAT: Fetal brown adipose tissue
GMP: Good manufacturing practices
H&E: Hematoxylin and eosin
hEP cells: Human embryonic progenitor cells
hES cells: Human embryonic stem cells
hPS cells: Human pluripotent stem cells
NMF: Non-negative matrix factorization
OXPHOS: Oxidative phosphorylation
qRT-PCR: Quantitative real-time PCR
RFUs: Relative fluorescence units
SAT: Subcutaneous adipose tissue
WAT: White adipose tissue
