# Supplementary figures and images for: Clonal derivation of white and brown adipocyte progenitor cell lines from human pluripotent stem cells
Source: Stem Cell Res Ther. 2019 Jan 8;10:7. doi: 10.1186/s13287-018-1087-7 (PMC6323697; doi:10.1186/s13287-018-1087-7)

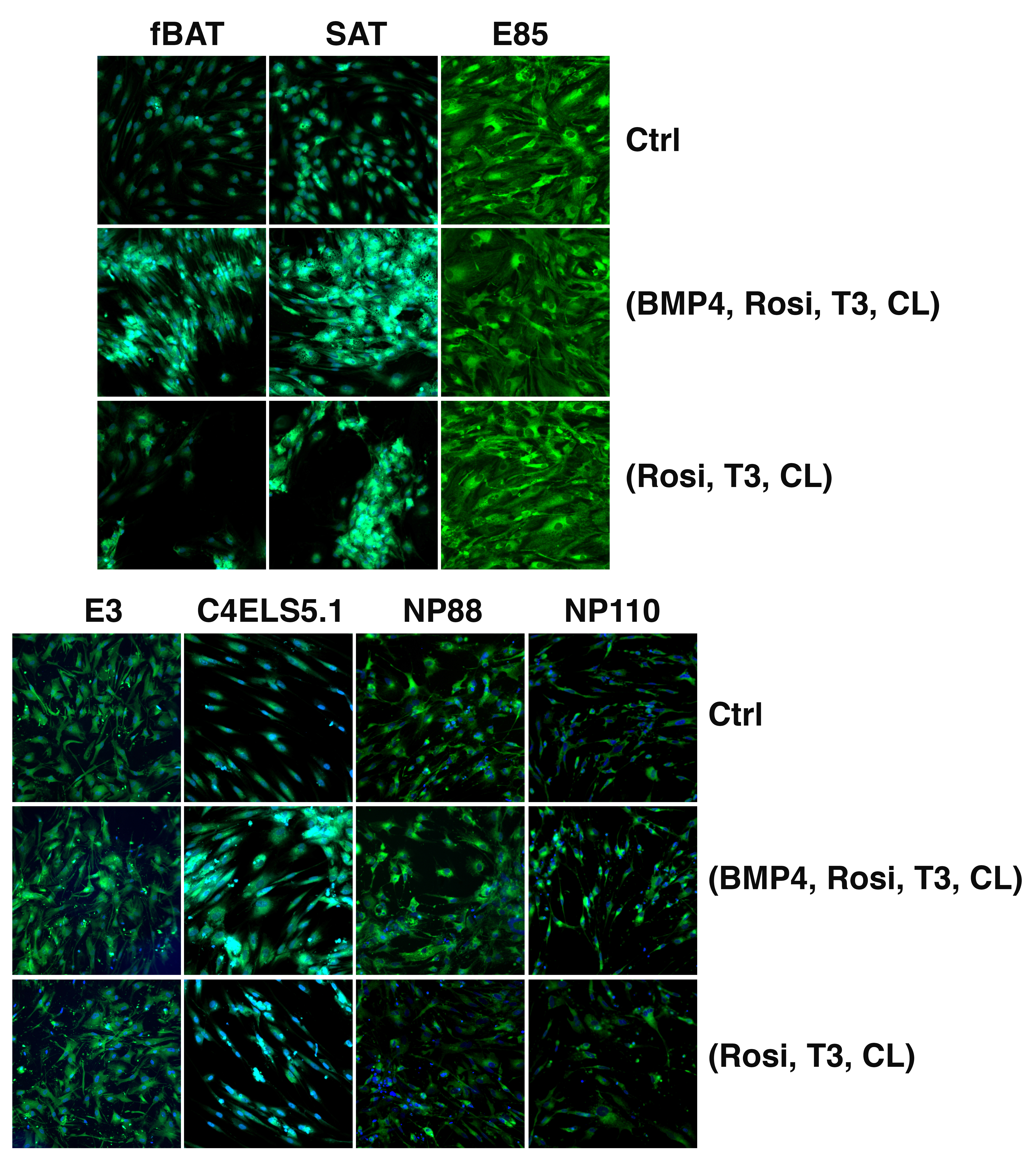

Supplement: Supplementary file 4 — Figure S2. Microscopic photographs of cells following mitochondrial staining in the progenitor state (Ctrl), in (BMP4, Rosi, T3, CL) and (Rosi, T3, CL) differentiation conditions. (JPG 8400 kb) [file 13287_2018_1087_MOESM4_ESM.jpg]

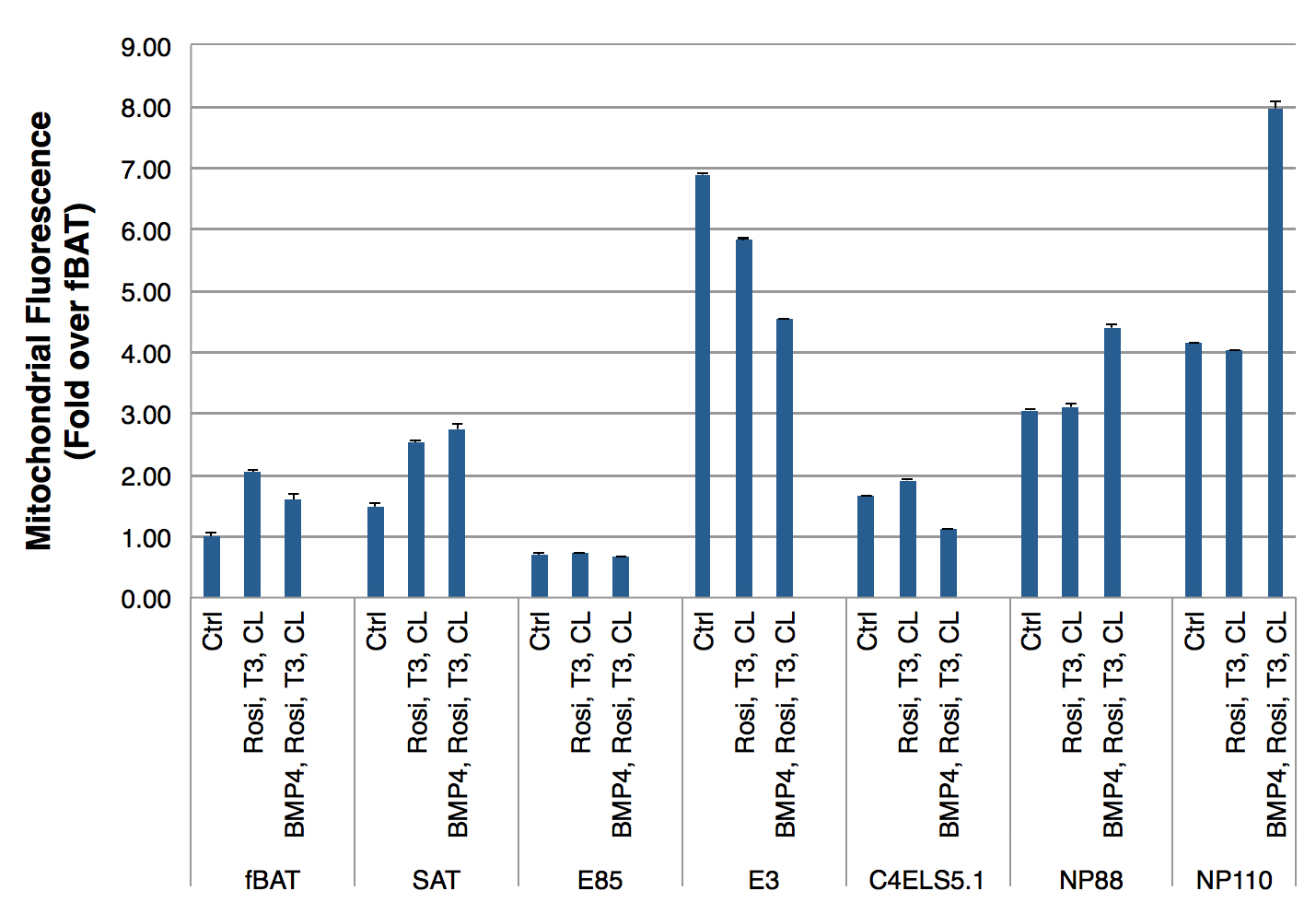

Supplement: Supplementary file 5 — Figure S3. Quantitation of mitochondria in the cells in the progenitor state (Ctrl), in (BMP4, Rosi, T3, CL) and (Rosi, T3, CL) differentiation conditions. (JPG 466 kb) [file 13287_2018_1087_MOESM5_ESM.jpg]

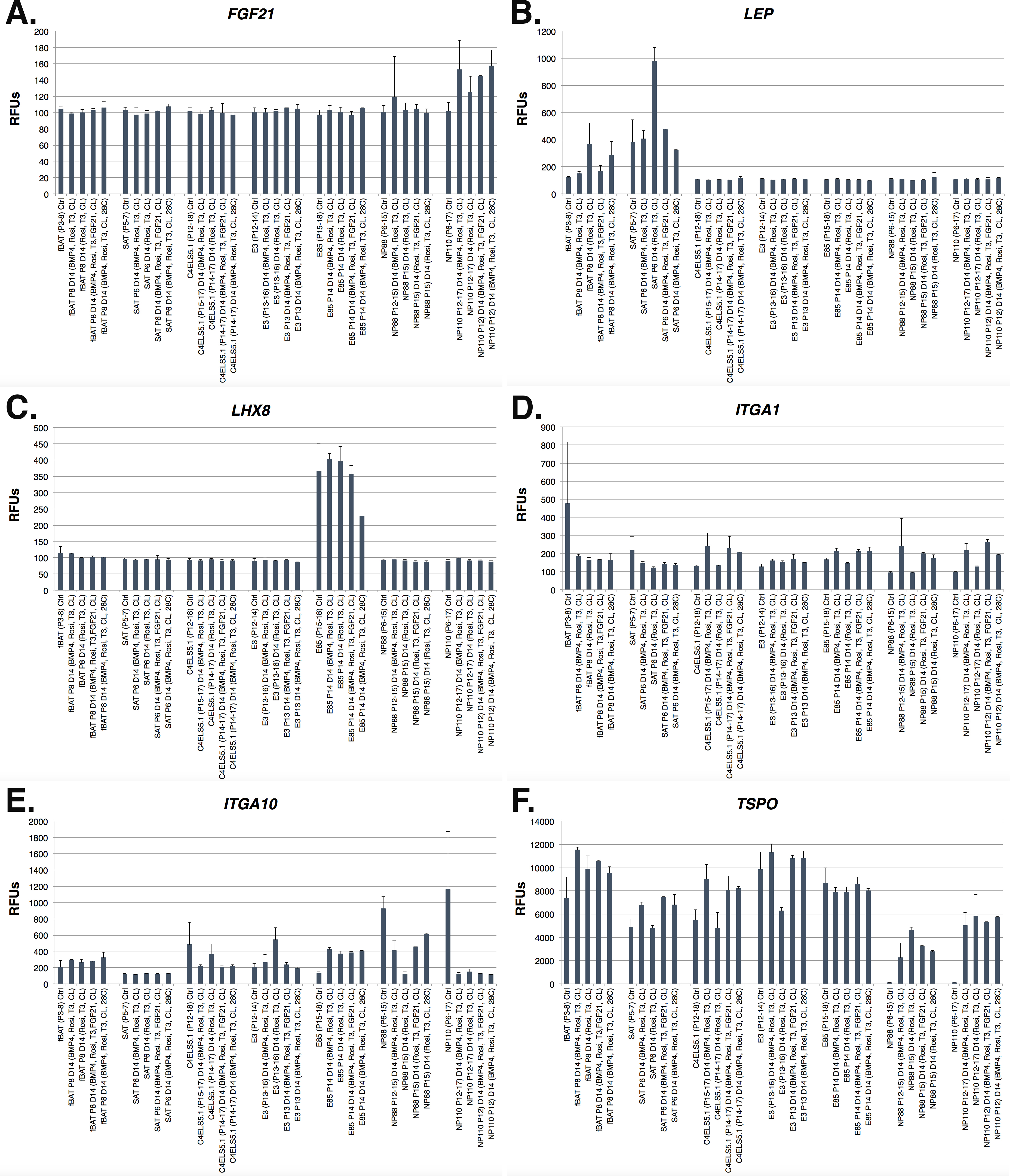

Supplement: Supplementary file 8 — Figure S4. Illumina-based RFU values for the expression of FGF21, LEP, LHX8, ITGA1, ITGA10, and TSPO in the lines under four diverse differentiation conditions. (JPG 6489 kb) [file 13287_2018_1087_MOESM8_ESM.jpg]

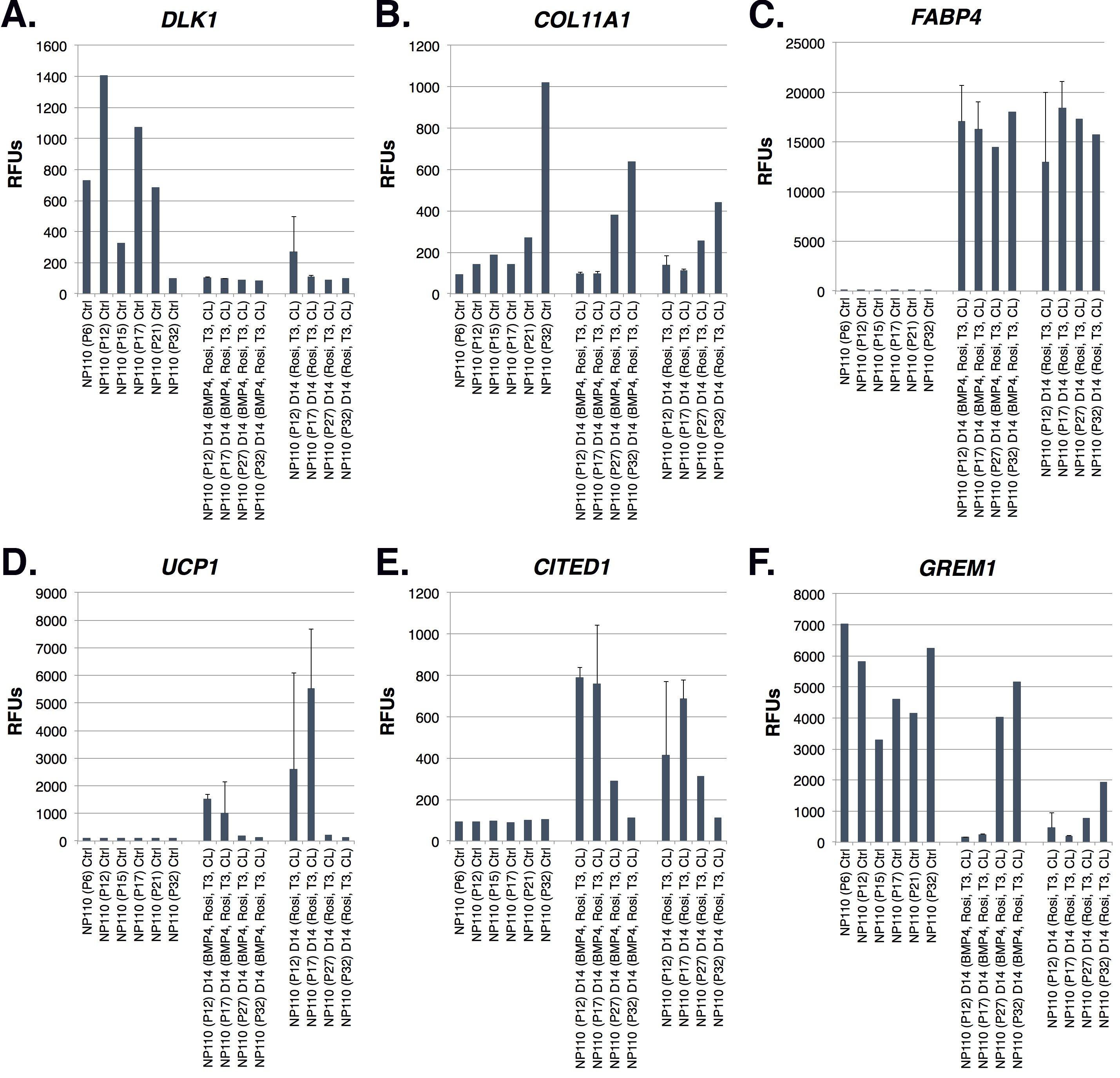

Supplement: Supplementary file 10 — Figure S5. Expression of select genes in the line NP110 at increasing passage numbers determined by Illumina Bead array. Cell line NP110 was cultured in D14 HyStem differentiation conditions with or without BMP4. Data are displayed as mean values (n = 3 ± standard deviation). (JPG 2816 kb) [file 13287_2018_1087_MOESM10_ESM.jpg]

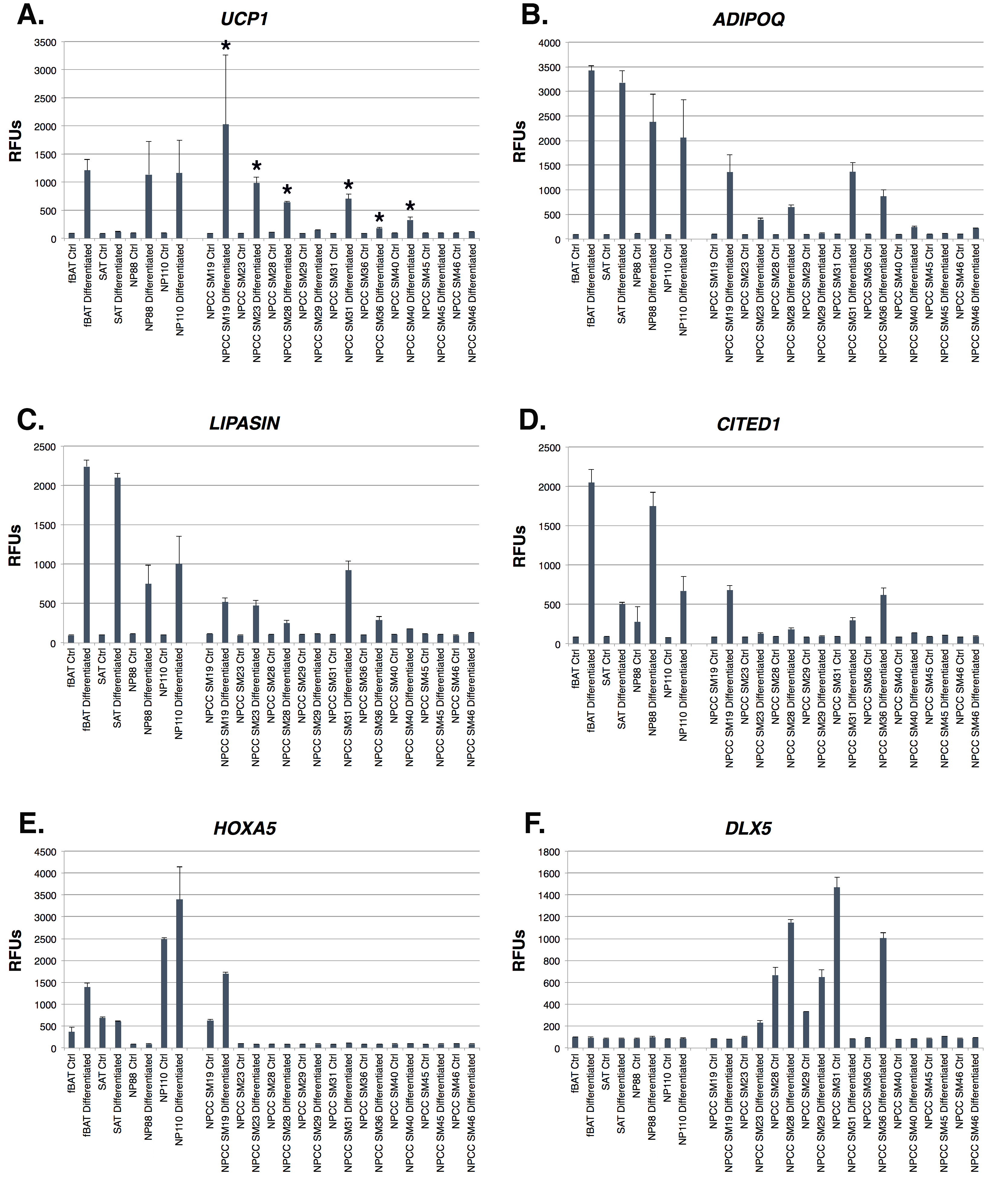

Supplement: Supplementary file 11 — Figure S6. BAT differentiation potential of clonal lines that were re-derived from candidate culture used to derive NP88 and NP110. Data are displayed as mean values of two or more biological replicates generated on Illumina gene expression bead arrays for (A) UCP1, (B) ADIPOQ, (C) LIPASIN, (D) CITED1, (E) HOXA5, and (F) DLX5. (*) marks clones designated clonal BAT progenitors. (RFU values < 130 considered background signal). (Error bars represent standard deviation). (JPG 4167 kb) [file 13287_2018_1087_MOESM11_ESM.jpg]
